# The Part and the Whole: Field‐Dependent THz‐EPR Spectroscopy Probes Lattice‐Dependent Electronic Structure in Iron(II) Spin Crossover

**DOI:** 10.1002/anie.4674308

**Published:** 2026-09-02

**Authors:** Jooris Beyer, Gerald Ebert, Andreas Dürrmann, Benjamin Kintzel, Gerald Hörner, Tarek Al Said, Karsten Holldack, Birgit Weber, Winfried Plass

**Affiliations:** ^1^ Institut für Anorganische und Analytische Chemie Friedrich‐Schiller‐Universität Jena Jena Germany; ^2^ Helmholtz‐Zentrum Berlin für Materialien und Energie Berlin Germany

**Keywords:** ab initio calculations, electronic structure, iron, magnetic properties, spin crossover, THz‐EPR spectroscopy

## Abstract

Frequency‐domain Fourier‐transform (FD‐FT) THz‐EPR spectroscopy provides direct access to kinetically trapped high‐spin (*HS*) states in iron(II) spin crossover (SCO) complexes. Using temperature‐induced excited spin‐state trapping (TIESST), the low‐temperature electronic structure of the *HS* state is accessed despite a diamagnetic low‐spin (*LS*) ground state. Field‐dependent spectra resolve the splitting of the *HS* ground‐state manifold and provide evidence for spin–phonon coupling, described by an avoided‐crossings Hamiltonian supported by ab initio calculations. THz‐EPR further quantifies the remaining *HS* fraction ($\gamma_{\text{HS}}$) and reveals shifts of magnetic transitions that depend on the surrounding spin‐state matrix. These results show that *HS* centers experience distinct local lattice environments in mixed *HS*/*LS* states and provide experimental access to the influence of spin‐state distribution on SCO processes. Cooperative switching scenarios, including domain formation and alternating *HS*/*LS* arrangements, can thus be distinguished. Field‐dependent THz‐EPR spectroscopy therefore provides direct access to lattice‐dependent effects in SCO materials.

## Graphical abstract

THz‐EPR spectroscopy provides direct access to the low‐lying electronic structure and spin–phonon coupling in iron(II) spin crossover (SCO) complexes. Quantification of the high‐spin fraction ($\gamma_{\text{HS}}$) and subtle shifts in magnetic transitions reveal the influence of the surrounding lattice and enable discrimination between microscopic switching pathways.

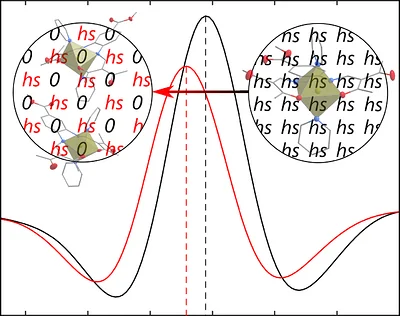

Molecular materials rely on interactions among their constituents that can give rise to emergent properties [1]. In turn, the blurred borderline between molecule and material requires the development of “emergent” methods, capable of analyzing the supramolecular interactions, which bring about cooperative effects.

Emergence of magnetic bistability is a desirable feature for creating switchable molecules [2]. Among its various manifestations, the spin crossover (SCO) phenomenon is especially promising for tunable and stimuli‐responsive switching. At the molecular level, SCO involves a change in multiplicity in certain 3d metal complexes—typically between high‐ (*HS*) and low‐spin (*LS*) states—which leads to pronounced changes in macroscopic physical properties [3]. Such systems can exhibit thermal hysteresis over a broad temperature range, including around or above room temperature. However, to fully exploit this potential in practical technologies [4, 5], a deeper understanding of the switching mechanism, in particular the origin of cooperativity at the supramolecular level, is required.

In this work, we introduce frequency‐domain Fourier‐transform (FD‐FT) THz‐EPR spectroscopy as an emergent analytical tool for studying cooperative phenomena in SCO materials. THz‐EPR is energetically located at the interface of local processes (zero‐field splitting states) and non‐local excitations (lattice phonons). As shown here, the method sensitively probes lattice effects on the multiplet structure of spin centers with high resolution.

Although quantum tunneling has been experimentally and theoretically identified as a possible pathway for SCO in isolated molecular species [6, 7], comprehensive experimental access to the cooperative mechanisms in bulk SCO solids—the transition from the part to the whole—remains limited despite theoretical advances [8], and often relies on real‐space imaging of large single crystals [9]. In this context, a variety of spectroscopic techniques have been employed to monitor the spin‐switching process, complementing SQUID magnetometry and x‐ray diffraction (Figure 1a) [10]. Multimethod approaches have provided insight into domain wall dynamics (ultrasound spectroscopy) [11], photo‐switching processes (x‐ray absorption) [12], and growth mechanisms [9].

**FIGURE 1:**
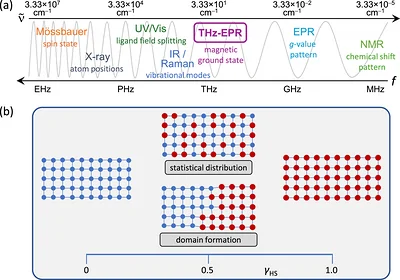
(a) Electromagnetic spectrum highlighting spectral regions accessible to techniques probing SCO phenomena; THz‐EPR at the interface of electronic and lattice excitations. (b) Schematic illustration of the spin‐state transition as a function of the high‐spin fraction $\gamma_{\text{HS}}$, indicating statistical distributions and domain formation.

UV–vis spectroscopy can deliver complementary information but lacks sensitivity to supramolecular aggregation [13]. Vibrational spectroscopies such as IR and Raman provide access to structural changes in the solid state [14, 15], yet the extraction of cooperativity‐relevant information is often non‐trivial [16]. NMR spectroscopy is largely restricted to solution studies and hampered by paramagnetic SCO‐active states [17, 18], although recent advances in solid‐state NMR have been reported [19]. EPR spectroscopy in the microwave regime can access paramagnetic states but is limited to a small subset of SCO‐active ions and suffers from line broadening in solids due to strong spin–spin interactions [20]. 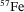 Mössbauer spectroscopy is sensitive to both local and non‐local effects in the solid [21], yet indirect probing of spin transitions does not readily yield microscopic structural information. Distinguishing between competing switching scenarios—such as domain formation versus statistical or low‐dimensional *HS*/*LS* distributions (Figure 1b)—thus remains beyond the reach of these established techniques.

Some SCO compounds have been investigated in the THz range [22, 23, 24, 25], and recent ab initio studies support the occurrence of spin–phonon coupling associated with the SCO process in the solid state [26, 27]. External magnetic fields are expected to reveal additional spectral information in this frequency regime. Accordingly, frequency‐domain THz‐EPR spectroscopy is particularly well suited to probe subtle energetic splittings within magnetic state manifolds [28, 29] and, especially in single‐molecule magnets, their coupling to lattice modes [30, 31].

Despite these advances, only few iron complexes have been studied to date [32, 33, 34], and none of them are SCO‐active. This is mainly due to the diamagnetic (S=0) ground state of iron(II) SCO complexes at low temperatures, which renders them silent in conventional THz‐EPR experiments.

To address this challenge, we investigate the kinetically active SCO compounds **1** and **2** using frequency‐domain Fourier‐transform (FD‐FT) THz‐EPR spectroscopy. Both systems were recently reported by some of us (Figure 2 and Supporting Information) [35, 36] and exhibit pronounced kinetic effects in their spin‐state switching behavior. Since the spin‐state dynamics of bulk complexes **1** and **2** between ca. 80 and 100 K proceed on timescales comparable to typical cooling rates of SQUID magnetometers, a kinetically trapped *HS* fraction ($\gamma_{\text{HS}}$) can be pre‐selectively under non‐equilibrium conditions. This enables direct access to trapped *HS* states and their comparison with relaxed configurations, providing a handle to probe different structural arrangements such as domain formation or alternating/statistical distributions.

**FIGURE 2:**
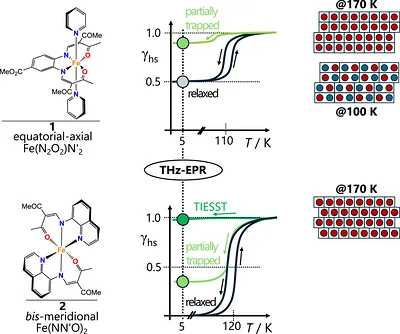
Molecular structures (left), SCO profiles (middle), and schematic representations of spin states in crystal structures (right) of complexes **1** [35] (top) and **2** [36] (bottom). Both complexes feature an octahedral $N_{4}O_{2}$ coordination sphere but differ in their ligand topologies.

The spin‐state switching in both **1** and **2** is governed by kinetic effects, allowing for the retention of a defined *HS* fraction $\gamma_{\text{HS}}$ at low temperatures via the temperature‐induced excited spin‐state trapping (TIESST) effect. By controlling the cooling conditions, $\gamma_{\text{HS}}$ can be tuned, providing a unique opportunity to investigate how the surrounding spin‐state matrix influences the properties of the residual *HS* centers. In the case of **1**, the fully relaxed low‐temperature structure is characterized by a 1:1 stoichiometry of *HS* and *LS* states ($\gamma_{\text{HS}}=0.5$), in which the spin states are arranged in alternating chains along the crystallographic [100] direction [35]. This well‐defined arrangement provides a structural benchmark that allows us to specifically probe the signatures of *HS* centers within a defined *LS* environment.

In the following, we first analyze the energetic splitting of the S=2 *HS* state of the iron(II) centers as revealed by combined theoretical and experimental data. In a second step, we exploit the variable, kinetically controlled spin‐state distribution to probe the influence of THz‐EPR‐silent *LS* states on the residual *HS* fraction—that is, the behavior of the part within the whole. This approach not only enables the determination of $\gamma_{\text{HS}}$ but also provides insight into the spin‐switching mechanism and the nature of the low‐temperature state based on characteristic spectroscopic signatures.

To this end, we consider the electronic structure of the *HS* states based on three distinct structural models derived from x‐ray diffraction (Figure S1): complex **1** in its pure *HS* state (at 170 K, denoted as **1** hs), the same complex within a mixed‐spin lattice (at 100 K, denoted as **1** hs@ls) [35], and complex **2** in its *HS* state (at 170 K, **2** hs) [36]. Ab initio calculations were performed using a state‐interaction approach including spin–orbit coupling (SOC‐SI). The primary analysis was conducted at the CASSCF/CASPT2/RASSI‐SO level (OpenMolcas 24.02) [37], and the robustness of the results was further validated by a CASSCF/NEVPT2/QDPT scheme (ORCA 6.0) [38] (see Supporting Information for details). Both methodologies yield highly consistent results regarding the ground‐state manifold and anisotropy parameters.

In all cases, a *HS* $d^{6}$ configuration with a quintet ground state (S=2) is obtained, while triplet and singlet states are well separated from the 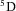 ground‐state manifold (Tables S1–S3). The 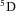 term splits into five non‐degenerate magnetic eigenstates ($m_{S}=\pm 2,\pm 1$, and 0), as depicted in Figure S5. Owing to strong spin–orbit coupling and state mixing $m_{S}$ is not a strictly good quantum number (Table S4). The first excited state within the 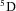 multiplet lies according to the ab initio calculations only 1 and 3 ${\mathrm{cm}}^{-1}$ above the ground state for **1** hs and **2** hs, respectively, followed by higher‐lying states at 16/17, 26/33, and 29/35 ${\mathrm{cm}}^{-1}$ (Table S3).

Effective spin Hamiltonian parameters (g, D, and E) were extracted from the SOC‐SI wavefunctions using the SINGLE_ANISO module [39] and the EPR/NMR module in ORCA, respectively. The calculations yield nearly isotropic values ($g_{\text{av}}=2.08$ for **1** hs, 2.10 for **2** hs) and moderate zero‐field splitting (ZFS) with pronounced rhombicity (E/D = 0.24 and 0.33, respectively, see Table S5). The consistency between the CASPT2(6,10) and NEVPT2(6,5) results confirms that the electronic structure and single‐ion anisotropy are robustly described. Overall, the calculated parameters are in good agreement with experiment (see below). Comparison of **1** hs and **1** hs@ls reveals only marginal changes (<1 ${\mathrm{cm}}^{-1}$) in the low‐energy level structure, which are expected to be experimentally inaccessible by conventional magnetic techniques, but become resolvable by THz‐EPR spectroscopy due to its direct energy sensitivity.

The analysis of THz‐EPR spectra is inherently linked to the paramagnetic electronic structure [40]. **2** provides an ideal model system, as its metastable *HS* state (hereafter denoted as *HS*‐t) can be trapped nearly quantitatively even at low temperatures using moderate cooling rates. In the kinetically trapped *HS*‐t state, $\gamma_{\text{HS}}$ reaches its maximum value for a given cooling protocol. In fact, rapid cooling (10–20 K/min) allows spectroscopic detection of the trapped *HS*‐t state at 5 K. The resulting magnetic‐field division spectra (MDS, obtained by dividing the transmittance recorded at $B_{0}+\Delta B$ by that at $B_{0}$, thereby suppressing field‐independent background and enhancing paramagnetic transitions) and temperature‐division spectra (TDS, obtained analogously from spectra at two different temperatures) are shown in Figure 3. Six transitions are observed in the MDS, exceeding the four transitions expected for a 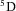 multiplet in which only the ground state is thermally populated. This arises because at 5 K the thermal energy ($k_{B}T\approx 3.5$ ${\mathrm{cm}}^{-1}$) exceeds the energy gap between the two lowest magnetic eigenstates (≈1 ${\mathrm{cm}}^{-1}$ for **2** as obtained from the experimental fit; the ab initio calculations yield ≈3 ${\mathrm{cm}}^{-1}$, Table S3), so that both states carry significant Boltzmann population (see Figure S5). Each of the two populated states gives rise to three observable transitions to the higher‐lying states 3, 4, and 5, yielding six features grouped into three pairs, where each pair is separated by a constant energy offset equal to this gap (see Table S6 and Supporting Information for assignments). Based on this information, the spectra were fitted using the spin Hamiltonian in Equation (1).

(1) $$\hat{H}=g\mu_{B}B\hat{S}+D\left[{\hat{S}}_{z}^{2}-\frac{1}{3}\hat{S}\left(\hat{S}+1\right)\right]+E\left({\hat{S}}_{x}^{2}-{\hat{S}}_{y}^{2}\right).$$

**FIGURE 3:**
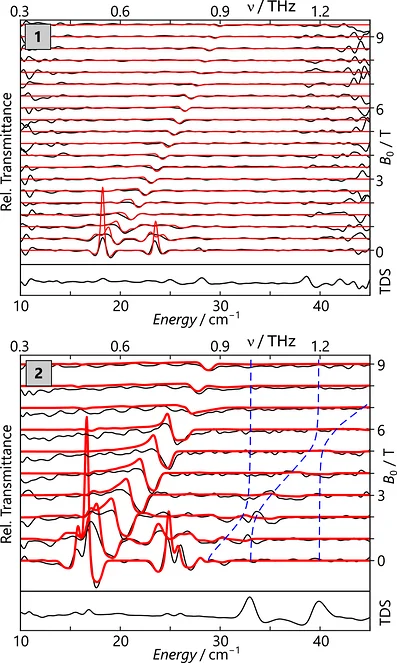
FD‐FT THz‐EPR results for **1** (upper panel) and **2** (lower panel): (top) Magnetic‐field division spectra (MDS) for fields ranging from 0 to 9 T ($\left(B_{0}+0.5\;T\right)/B_{0}$ for **1**, $\left(B_{0}+1\;T\right)/B_{0}$ for **2**, at 5 K) and (bottom) temperature‐division spectra (TDS) with 20 K/10 K for **2**, 20 K/5 K for **1**, at 0 T. Black lines represent the experimental data, red lines the best fits to Equation (1) using the parameters given in Table 1, and dashed blue lines the results including spin–phonon coupling (Equation S1). Spectra were recorded using a mercury lamp as light source.

The Hamiltonian comprises an isotropic g value and axial (D) and rhombic (E) zero‐field splitting terms. The resulting parameters (Table 1) reproduce the experimental spectra well (Figures 3 and S6) and confirm the pronounced rhombicity predicted by theory. Deviations for **2** at higher energies (at ≈ 33 and 40 ${\mathrm{cm}}^{-1}$) indicate spin–phonon coupling, which can be reproduced using an extended Hamiltonian (Equation S1), yielding good agreement with the observed spectral features (Figure 4). These results demonstrate that TIESST enables direct probing of the *HS* electronic structure of iron(II) SCO complexes with diamagnetic ground states by THz‐EPR spectroscopy.

**TABLE 1:** Parameters from the best fit of the MDS to the Hamiltonian given in Equation 1 for **1** and **2** with S=2.

	$g_{\text{iso}}$	|D| / ${\mathrm{cm}}^{-1}$	|E| / ${\mathrm{cm}}^{-1}$	E/D
**1**	2.088	6.915	0.896	0.130
**2**	2.088	6.759	1.515	0.224

**FIGURE 4:**
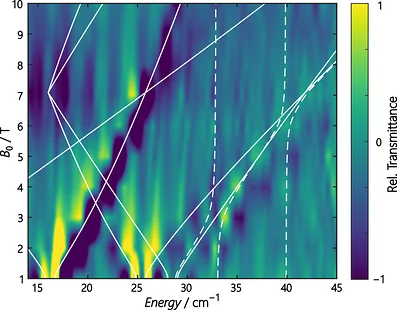
Contour plots of the magnetic‐field division spectra (MDS, $\left(B_{0}+1\;T\right)/B_{0}$, 5 K) of **2**. Relative intensity is color‐coded as indicated by the scale bar. Solid white lines show the simulated signal shifts from the best fit to Equation 1, while dashed white lines include spin–phonon coupling (Equation S1). Spectra were recorded using a mercury lamp as light source.

With direct access to the trapped *HS*‐t state at low temperature (5 K), relaxation to the *LS* state was induced by heating the sample to temperatures close to or slightly above $T_{\text{TIESST}}$ [35, 36]. Upon thermal relaxation and subsequent cooling, the system reaches a relaxed *LS* state (*LS*‐r) or a mixed‐spin state with a residual *HS* fraction ($\gamma_{\text{HS}}$). The temperature cycle comprised both temperature‐ and time‐dependent elements, combining field‐dependent THz‐EPR measurements at low temperatures with time‐resolved spectra recorded at elevated temperatures without magnetic field variation (see Supporting Information for details on the measurement protocols).

While magnetic transitions are only directly observable at low temperatures in field‐dependent THz‐EPR measurements, spectra recorded at elevated temperatures at zero magnetic field provide access to the evolution of the spin‐state population via changes in spectral intensity. In particular, consecutive spectra recorded under quasi‐isothermal conditions at elevated temperatures reveal a gradual change in intensity, reflecting the relaxation of the kinetically trapped *HS*‐t fraction. This behavior is consistent with a thermally activated SCO process accompanied by structural and vibrational changes in the lattice [27].

After prolonged annealing at elevated temperature (Figure S7), no further spectral changes were detected, indicating that a quasi‐stationary state had been reached. The sample was then rapidly cooled back to 5 K, where paramagnetic THz‐EPR signals with reduced intensity were observed, corresponding to a residual *HS* fraction in a relaxed matrix (*LS*‐r), demonstrating that a fraction of iron(II) centers remains in the *HS* state (Figure S8).

By referencing the signal intensity to the fully trapped *HS*‐t state (obtained by rapid cooling), the remaining *HS* fraction $\gamma_{\text{HS}}$ can be quantified. For **2**, this analysis yields $\gamma_{\text{HS}}\approx 0.33$, in good agreement with the value obtained from slow‐cooling experiments (0.5 K/min, $\gamma_{\text{HS}}=0.36$) [36].

Although the experimental setup does not allow for the precise control of cooling rates achievable in SQUID magnetometry, THz‐EPR spectroscopy thus provides a direct and independent estimate of $\gamma_{\text{HS}}$. To the best of our knowledge, this represents the first quantitative determination of $\gamma_{\text{HS}}$ by THz‐EPR spectroscopy.

Beyond the intensity changes used for quantification, the THz‐EPR spectra of **1** exhibit subtle but systematic shifts in the transition energies upon spin crossover (Figure 5). Comparison of spectra recorded before ($E_{\text{ante}}$) and after ($E_{\text{post}}$) thermal relaxation reveals well‐defined energy shifts of $\Delta E_{1}\left(1\right)=0.12\;{\text{cm}}^{-1}$ and $\Delta E_{2}\left(1\right)=-0.15\;{\text{cm}}^{-1}$. Importantly, these shifts are consistently observed across independent measurements using different radiation sources (mercury lamp and synchrotron radiation), demonstrating that they represent an intrinsic property of the system rather than an experimental artifact. The observation that spectra of comparable quality are obtained with a conventional mercury lamp demonstrates that, for compounds with magnetic transitions in the mid‐to‐far THz range (as in the present case, where transitions exceed ∼15 ${\mathrm{cm}}^{-1}$), the technique is accessible to standard laboratory settings without requiring dedicated synchrotron facilities. A direct experimental quantification of the ZFS change is provided by field‐dependent MDS measurements of the post‐SCO state of **1**, recorded over the range 0–4 T and fitted independently to Equation (1), yielding post‐state parameters g=1.94, $\left|D\right|=6.915\;{\text{cm}}^{-1}$, $\left|E\right|=0.827\;{\text{cm}}^{-1}$; fits and parameters are presented in the Supporting Information (Figure S13 and Table S7). Comparison with the ante‐state values (Table 1) yields $\Delta |D|\approx 0\;{\text{cm}}^{-1}$ and $\Delta |E|=-0.069\;{\text{cm}}^{-1}$, that is, a decrease in rhombicity. Both the sign and order of magnitude of Δ|E| are consistent with the ab initio prediction for the 1hs →1hs@ls structural transition ($\Delta |E|=-0.12\;{\text{cm}}^{-1}$, from |E|=1.60 to $1.48\;{\text{cm}}^{-1}$, Table S5), confirming that the energy shifts reflect the specific crystallographic *HS*/*LS* ordering. The unchanged |D| is also consistent with theory ($\Delta D=-0.07\;{\text{cm}}^{-1}$, within fitting uncertainty at this scale). The slight decrease in g (Δg=−0.15), which is not expected from theory, is attributed to the limited field range of the post‐state data (0–4 T vs. 0–9 T for the ante state), which constrains the determination of g less well.

**FIGURE 5:**
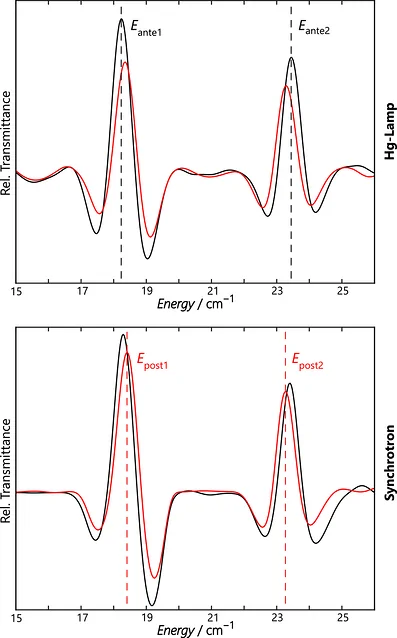
Magnetic‐field division spectra (MDS, (1 T)/(0 T), 5 K) of **1** before (black lines) and after (red lines) thermally induced spin crossover. Top: spectra recorded using a mercury lamp; bottom: spectra recorded using synchrotron radiation on a separate pellet. Dashed lines indicate the positions of selected transitions before (top, $E_{\text{ante}}$) and after (bottom, $E_{\text{post}}$) spin crossover.

The observed energy shifts indicate a modification of the electronic structure of the remaining *HS* centers upon transformation of the surrounding spin‐state matrix. While the primary coordination sphere of the iron(II) ions remains essentially unchanged, the transition from a predominantly *HS* lattice to a mixed *HS*/*LS* environment alters the second coordination sphere. This modification perturbs the local electronic structure of the *HS* sites via changes in electrostatic and lattice interactions, resulting in measurable changes of the zero‐field splitting.

For **1**, this effect can be interpreted unambiguously, as the low‐temperature structure is well established. The spin crossover leads to a defined mixed *HS*/*LS* state with an ordered arrangement of alternating columns and a fixed $\gamma_{\text{HS}}$ of 0.5 [35]. Consequently, the change in the local environment of the *HS* centers upon relaxation is well defined, and the resulting energy shifts are reproducible in both magnitude and sign.

These observations demonstrate that even subtle lattice modifications—while preserving the primary coordination sphere—translate into measurable changes in the zero‐field splitting. THz‐EPR spectroscopy thus provides direct access to the local environment of paramagnetic centers within a mixed spin‐state matrix.

This sensitivity enables discrimination between different microscopic spin‐crossover pathways. In a scenario involving three‐dimensional domain growth, most *HS* centers would experience a homogeneous environment, and only molecules at domain boundaries would be affected, resulting in negligible overall spectral shifts. In contrast, statistical distributions or low‐dimensional ordering motifs inherently modify the local environment of a large fraction of *HS* sites, leading to the experimentally observed energy shifts (cf. Figure 1b). Crucially, the magnitude and pattern of the observed shifts are quantitatively reproduced by the ab initio prediction for the specific crystallographic *HS*/*LS* arrangement of **1** (1hs@ls, Table S5), confirming that THz‐EPR detects a well‐defined structural signature rather than a generic environmental perturbation. This mechanistic discrimination—between domain growth and statistical or low‐dimensional ordering—is inaccessible to existing bulk techniques such as SQUID magnetometry or x‐ray diffraction, and constitutes the principal advance demonstrated here.

The behavior of **2** can be rationalized within the same framework (Figure 6). Upon spin crossover, the transition energies exhibit shifts of 0.02–0.12 ${\mathrm{cm}}^{-1}$, which are qualitatively reproducible across independent measurements, including experiments performed with synchrotron radiation. However, in contrast to **1**, a quantitative interpretation is hampered by the lack of structural information on the low‐temperature *LS* state and by the presence of kinetically controlled *HS*/*LS* mixtures with variable $\gamma_{\text{HS}}$. As a result, the local environment of the *HS* centers is not uniquely defined.

**FIGURE 6:**
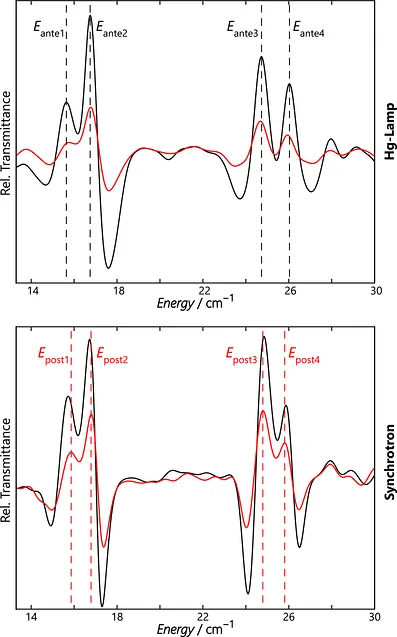
Magnetic‐field division spectra (MDS, (1 T)/(0 T), 5 K) of **2** before (black lines) and after (red lines) thermally induced spin crossover. Top: spectra recorded using a mercury lamp; bottom: spectra recorded using synchrotron radiation on a separate pellet. Dashed lines indicate the positions of selected transitions before (top, $E_{\text{ante}}$) and after (bottom, $E_{\text{post}}$) spin crossover.

Despite these limitations, the consistent observation of energy shifts in **2** indicates that *HS* centers are embedded in a varying *LS* matrix, pointing toward a statistical or low‐dimensional mechanism for the kinetic relaxation of the TIESST‐trapped state. We note that this conclusion does not necessarily extend to the thermodynamic SCO transition of **2** at elevated temperatures, which may proceed via a more cooperative, domain‐driven pathway, as documented for related systems by optical microscopy [41, 42]. Variations in the absolute magnitude of the shifts reflect differences in $\gamma_{\text{HS}}$ and structural arrangement, while their consistent qualitative behavior supports a common underlying mechanism. Taken together, these results demonstrate that THz‐EPR spectroscopy provides direct spectroscopic access to the microscopic nature of cooperative spin‐state switching in the solid state.

In summary, the potential of FD‐FT THz‐EPR spectroscopy is demonstrated for two octahedral iron(II) SCO complexes. By exploiting the TIESST effect, direct access to the electronic structure of the trapped high‐spin (*HS*) state is achieved, including resolution of the ground‐state multiplet and identification of spin–phonon coupling. Beyond this, THz‐EPR spectroscopy enables quantitative determination of the remaining high‐spin fraction $\gamma_{\text{HS}}$ and, most importantly, directly discriminates between competing microscopic SCO mechanisms—three‐dimensional domain growth versus statistical or low‐dimensional ordering—based on measurable, lattice‐dependent changes in the electronic structure of residual *HS* centers.

The observation of systematic energy shifts demonstrates that residual *HS* centers reside in a modified lattice environment within a mixed *HS*/*LS* state. Using **1** as a well‐defined structural reference, this allows us to attribute the behavior of **2** to a locally intermixed, statistically distributed or low‐dimensionally correlated spin‐state arrangement, while excluding a mechanism dominated by three‐dimensional domain growth as well as contributions from SCO‐inactive phases.

These results establish THz‐EPR spectroscopy as a powerful tool to probe lattice‐dependent effects in SCO materials and to distinguish between competing switching pathways based on subtle changes in magnetic excitation spectra. While demonstrated here for TIESST‐active systems, the approach is expected to be applicable more broadly to SCO materials with incomplete or heterogeneous switching behavior. Future studies targeting systems with well‐defined domain‐driven transitions will further elucidate the relationship between lattice organization and spin‐state dynamics in cooperative switching materials.

## Conflicts of Interest

The authors declare no conflicts of interest.

## Supporting information


**Supporting File**: anie74350‐sup‐0001‐SuppMat.pdf.

## Data Availability

The data that support the findings of this study are available in the Supporting Information of this article.
