# Supplementary material for: The Part and the Whole: Field‐Dependent THz‐EPR Spectroscopy Probes Lattice‐Dependent Electronic Structure in Iron(II) Spin Crossover
Source: Angew Chem Int Ed Engl. 2026 Sep 2;65(42):e4674308. doi: 10.1002/anie.4674308 (PMC13653631; doi:10.1002/anie.4674308)
Supplement: Supplementary file 1 [file ANIE-65-e4674308-s001.pdf]

Supporting Information

The Part and the Whole: Field-Dependent THz-EPR Spectroscopy Probes Lattice-Dependent Electronic Structure in Iron(II) Spin Crossover

Jooris Beyer, Gerald Ebert, Andreas Dürrmann, Benjamin Kintzel, Gerald Hörner, Tarek Al Said, Karsten Holldack, Birgit Weber,* and Winfried Plass*

Contents

S1. Ab Initio Calculations **S2**

S2. THz-EPR Measurements **S11**
S2.1. Experimental S11
S2.2. Low-Lying Electronic Structure S11
S2.3. Spin–Phonon Coupling S13
S2.4. Temperature- and Time-Dependent Measurements S13
S2.4.1. Time-Dependent Measurements at Elevated Temperatures S13
S2.4.2. Comparison of *HS*-t and *LS*-r States at 5 K S15
S2.4.3. Temperature-Dependent Measurements S16
S2.5. Field-Dependent MDS of the Post-SCO State of Compound **1** S21

List of Figures

S1. Structural models used for the ab initio calculations S3
S2. Orientation of the magnetic axes for **1**HS. S8
S3. Orientation of the magnetic axes for **1**HS@LS. S9
S4. Orientation of the magnetic axes for **2**HS. S10
S5. Five magnetic eigenstates of the $S = 2$ ground state ($^5T_{2g}$) in a distorted octahedral ligand field . . S11
S6. Contour plot of the MDS of **1** S12
S7. Time-dependent THz spectra of **1** and **2** S14
S8. THz spectra *HS*-t and *LS*-r states of **1** and **2** S15
S9. Temperature dependence of THz spectra for **1** S17
S10. Temperature dependence of THz spectra for **2** S18
S11. Temperature-dependent THz spectra of **1** S19
S12. Temperature-dependent THz spectra of **2** S20
S13. MDS of **1** post-SCO with EasySpin fit S21

List of Tables

S1. Relative energies from CASSCF calculations S4
S2. Relative energies including dynamic correlation S5
S3. Relative energies including spin–orbit coupling S6
S4. Projections of the magnetic eigenstates onto the m_S basis S6
S5. Calculated magnetochemical parameters for the structural models of **1** and **2**. S7
S6. Assignment of THz-EPR transitions of **2**HS S12
S7. Ante- and post-state ZFS parameters for **1** S21

S1. Ab Initio Calculations

All calculations were performed on structural models derived from the experimental single-crystal structures of **1**[1] and **2**.[2] Hydrogen atom positions were optimized using the Turbomole 7.8 package[3,4] at the RI-DFT[5–8]/PBE0[9,10]/def2-TZVPP[11] level of theory. For geometry optimizations, paramagnetic iron(II) ions were replaced by diamagnetic zinc(II) ions. The derived structural models for the high-spin (*HS*) iron(II) centers in the crystal structures of **1** and **2** are depicted in Figure S1 and denoted as **1**HS (crystal structure of **1** at 170 K, all iron(II) centers in the *HS* state) and **1**HS@LS (crystal structure of **1** at 100 K, mixed *HS/LS* states with $\gamma_{\mathrm{HS}} = 0.5$), as well as **2**HS (crystal structure of **2** at 170 K, all iron(II) centers in the *HS* state).

Single-ion magnetic anisotropy parameters were obtained via multi-reference CASSCF/CASPT2/RASSI-SO calculations as implemented in OpenMolcas 24.02.[12]. Relativistic ANO-RCC basis sets (iron and donor atoms: ANO-RCC-VTZP; remaining atoms: ANO-RCC-VDZP)[13] were employed in combination with a scalar-relativistic second-order Douglas-Kroll-Hess Hamiltonian. Cholesky decomposition of two-electron integrals was used (integral threshold: 10^{-8} a.u.). The CASSCF active space comprised 6 electrons in 10 orbitals (3d and 4d shells), accounting for 5 quintet (^{5}D), 45 triplet (^{3}P, ^{3}H, ^{3}F, ^{3}G, ^{3}D, ^{3}P, ^{3}F) and 50 singlet states (^{1}I, ^{1}G, ^{1}S, ^{1}D, ^{1}F, ^{1}P, ^{1}F, ^{1}G), results are summarized in Table S1. Dynamic correlation was subsequently treated using CASPT2 in its multi-state formulation[14] for all quintet (^{5}D), the 17 lowest triplet (^{3}P, ^{3}H, ^{3}F) and the 17 lowest singlet (^{1}I, ^{1}G) states (see Table S2). Spin–orbit coupling (SOC) and state mixing were addressed via RASSI-SO calculations. The resulting energy splittings are summarized in Table S3, while the composition of the five lowest-lying magnetic eigenstates into m_{S} levels of the $S = 2$ manifold is given in Table S4.[15,16] Magnetic parameters, such as the g and zero-field splitting (ZFS) tensors, were extracted using the SINGLE_ANISO module; results are summarized in Table S5.[17] The resulting magnetic axes are depicted in Figures S2–S4.

To validate the robustness of the calculated magnetic parameters against the choice of the perturbation theory variant and the active space size, an additional series of calculations was performed using the NEVPT2 method as implemented in ORCA 6.0.[18] While the reference CASPT2 calculations utilized a (6,10) active space to account for double-shell effects, the NEVPT2 calculations employed a more compact (6,5) active space (see Tables S1 and S2). The same set of electronic states as in the CASPT2 calculations was included, *i.e.*, 5 quintet, 45 triplet, and 50 singlet states arising from the d^6 configuration of iron(II) (see above), thus preserving the full multiplet structure and ensuring direct comparability between both approaches. These calculations were carried out at the RI-DKH2 level of theory using def2-SVP basis sets, with the larger def2-TZVP basis set assigned to the iron center and its immediate donor atoms. The RIJCOSX approximation was used to accelerate the treatment of two-electron integrals. Consistent with the RASSI-SO approach, SOC was treated by diagonalizing the SOC operator in the basis of the NEVPT2 states using quasi-degenerate perturbation theory (QDPT). The spin–orbit mean-field (SOMF) operator was employed to evaluate the SOC matrix elements. Magnetic parameters were subsequently extracted using the EPR/NMR module in ORCA, following a protocol analogous to the SINGLE_ANISO approach (see Table S3).

The comparison, summarized in Table S5, reveals a high degree of consistency between both methodologies. The deviations in the D and E parameters remain within a tolerable range of approximately 10–15%, with both methods yielding identical signs for the axial anisotropy. This agreement confirms that the simplified NEVPT2(6,5) scheme provides a qualitatively and quantitatively reliable description of the electronic structure and the resulting single-ion anisotropy in these complexes.

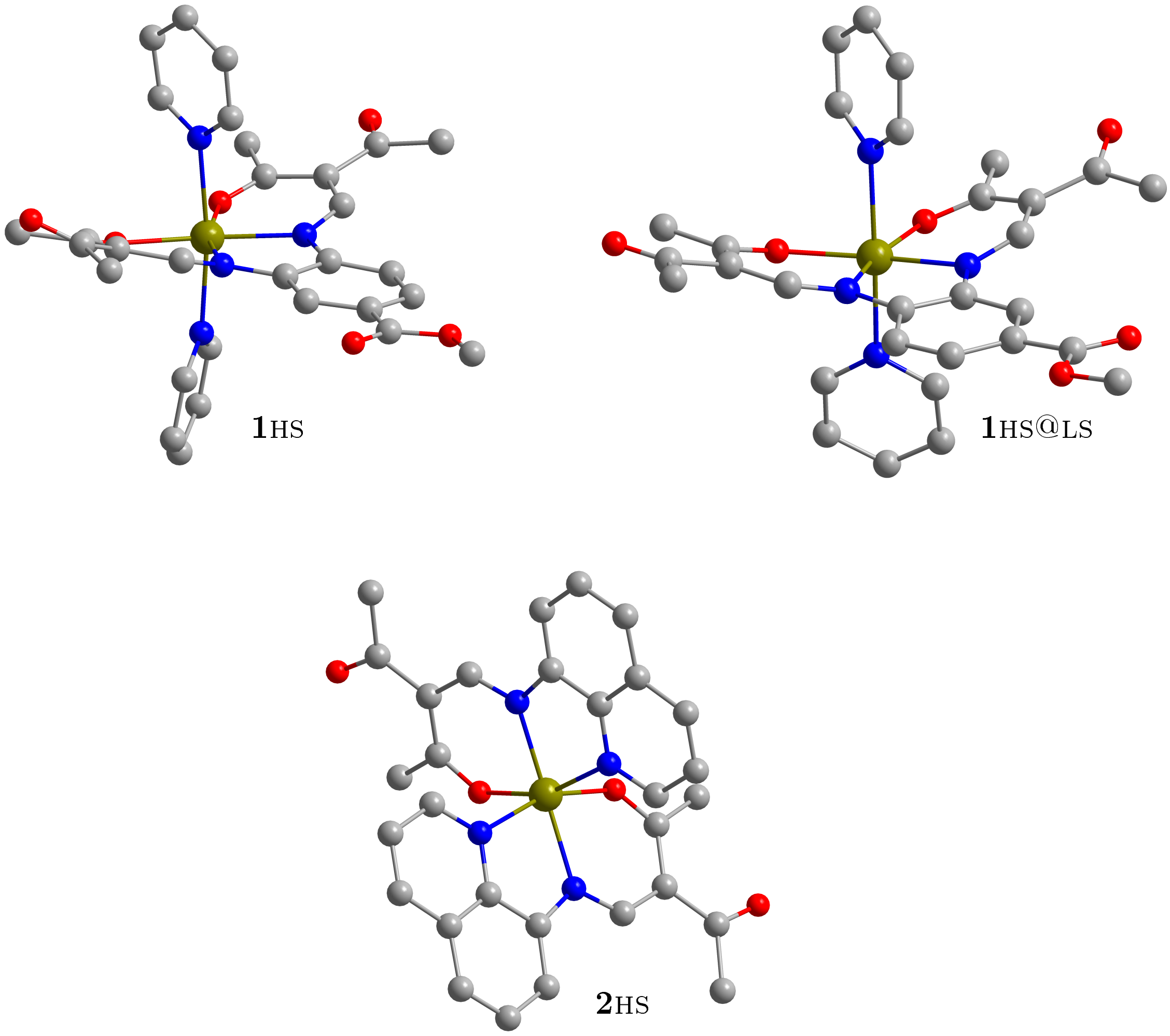

Figure S1. Structural models used for the ab initio calculations: **1**HS (top left), **1**HS@LS (top right), and **2**HS (bottom). Hydrogen atoms are omitted for clarity. Color code: iron (olive), oxygen (red), nitrogen (blue), and carbon (gray).

Table S1. Relative energies of the lowest electronic states at the CASSCF level, comparing (6,10) and (6,5) active spaces using OpenMolcas and ORCA, respectively.

ΔE / cm^{-1}	**1**HS		**1**HS@LS		**2**HS	
	(6,10)	(6,5)	(6,10)	(6,5)	(6,10)	(6,5)
$2S+1=5$ (*HS*)	0	0	0	0	0	0
	1630	1509	1726	1602	1371	1267
	2303	2165	2264	2120	1417	1296
	10135	9277	10508	9623	10627	9720
	13097	12110	13317	12313	11384	10447
$2S+1=3$ (*IS*)	14829	15596	14603	15380	15119	15892
	15268	15949	15089	15771	15377	16131
	16070	16802	15823	16568	15454	16141
	17997	18760	17900	18668	18588	19174
	19681	20134	19502	19970	19310	19922
	20592	21042	20419	20887	19725	20245
	22708	22943	22723	22971	22635	22891
	22829	22968	22873	22982	22937	23118
	23829	23980	23879	24024	23406	23536
	24896	25358	24835	25288	24622	25028
	25657	25943	25661	25923	25285	25524
	25726	26047	25818	26089	25714	26002
	25902	26093	25915	26166	25917	26236
	26977	27152	27011	27181	26273	26471
	27016	27182	27038	27193	26781	26944
	27651	27833	27645	27832	26868	27051
	28151	28314	28165	28321	27266	27414
$2S+1=1$ (*LS*)	17383	18897	16891	18430	17370	18911
	24225	25189	23973	24940	24447	25401
	24864	25751	24641	25531	24792	25718
	25378	26268	25120	26023	24881	25773
	31191	31781	31157	31745	30816	31404
	31420	31979	31311	31887	31117	31636
	31601	32108	31543	32041	31595	32106
	33061	33492	33118	33536	33774	34183
	34731	35441	34760	35468	34201	34569
	35194	35583	35208	35575	34977	35474
	35678	36074	35696	36103	36104	36673
	36920	37317	37037	37425	36307	36902
	37439	37938	37394	37934	37081	37560
	38623	38823	38701	38944	37619	37864
	38711	38939	38833	38999	38147	38409
	39099	39489	39086	39491	38572	38811
	39417	39622	39482	39667	38893	39058

Table S2. Relative energies of the lowest electronic states including dynamic correlation (CASPT2 and NEVPT2), based on the (6,10) and (6,5) reference wave functions using OpenMolcas and ORCA, respectively.

ΔE / cm^{-1}	**1**HS		**1**HS@LS		**2**HS	
	CASPT2	NEVPT2	CASPT2	NEVPT2	CASPT2	NEVPT2
$2S+1=5$ (HS)	0	0	0	0	0	0
	1760	1873	1867	1967	1570	1692
	2635	2784	2589	2733	1689	1790
	11052	11580	11467	12005	11511	12004
	14128	14798	14377	15051	12377	12905
$2S+1=3$ (IS)	12713	11046	12433	10759	12395	11813
	13282	11613	13044	11376	12659	12145
	14050	12345	13774	12014	13223	12337
	14595	14393	14442	14245	15786	16180
	17495	16906	17232	16628	16396	16928
	18103	17947	17852	17684	16897	17606
	20095	20731	20094	20751	20100	21224
	21262	20677	21284	20781	20473	21607
	21402	21968	21435	22002	20978	22179
	21226	21902	21159	21842	21108	22656
	22401	23443	22383	23515	22281	23802
	22496	23412	22492	23392	22602	24360
	23148	23771	22869	23756	22631	24487
	23627	24486	23690	24402	23211	24221
	24118	24754	24314	24820	23636	24542
	25023	26018	24982	25998	24185	25680
	24969	25546	24968	25551	24037	25182
$2S+1=1$ (LS)	14264	11485	13678	10856	14344	12222
	20581	20173	20258	19861	20298	21314
	21236	20894	20961	20608	20827	21695
	21918	21284	21592	20944	21142	21793
	26955	28261	26825	28223	26868	28833
	27528	28449	27283	28209	27285	29446
	27736	28761	27557	28707	27837	29972
	29751	30423	29764	30483	29500	31815
	30616	30706	30672	30890	30393	32177
	31352	32909	31124	32684	30426	33223
	32652	32839	32144	32855	31559	33262
	32436	34101	32347	34170	31307	33217
	32602	34172	32160	33863	33009	34297
	35239	36646	35063	36322	33959	36265
	35423	36075	35704	36625	33764	36590
	34768	36074	34701	35771	34473	37161
	35916	37239	36023	37520	35380	38104

Table S3. Relative energies of the magnetic eigenstates obtained from spin–orbit state-interaction calculations (SOC-SI) based on CASPT2 and NEVPT2 states using OpenMolcas and ORCA, respectively.

ΔE / cm^{-1}	**1**HS		**1**HS@LS		**2**HS	
	CASPT2	NEVPT2	CASPT2	NEVPT2	CASPT2	NEVPT2
State #						
1	0	0	0	0	0	0
2	1	1	1	1	3	3
3	16	18	16	19	17	19
4	26	29	25	29	33	37
5	29	33	29	33	35	39
6	1765	1880	1869	1970	1521	1631
7	1768	1882	1872	1972	1522	1632
8	1793	1911	1898	2002	1578	1696
9	1813	1930	1917	2021	1602	1722
10	1817	1936	1922	2027	1626	1752
11	2658	2811	2612	2760	1745	1851
12	2658	2811	2612	2760	1768	1879

Table S4. Percentage projections of the five lowest-lying magnetic eigenstates (see Table S3) onto the $|S=2, m_\mathrm{S}\rangle$ basis functions (denoted as $|m_\mathrm{S}\rangle$) of the ground-state manifold for **1**HS, **1**HS@LS, and **2**HS, as derived from RASSI-SO calculations using OpenMolcas (all values in %).

State #	**1**HS	**1**HS@LS	**2**HS
1	0.8 $|-2\rangle$	1.5 $|-2\rangle$	12.8 $|-2\rangle$
	49.0 $|-1\rangle$	48.0 $|-1\rangle$	2.9 $|-1\rangle$
	0.4 $|0\rangle$	1.0 $|0\rangle$	68.7 $|0\rangle$
	49.0 $|1\rangle$	48.0 $|1\rangle$	2.9 $|1\rangle$
	0.8 $|2\rangle$	1.5 $|2\rangle$	12.8 $|2\rangle$
2	14.6 $|-2\rangle$	14.8 $|-2\rangle$	2.5 $|-2\rangle$
	0.9 $|-1\rangle$	1.7 $|-1\rangle$	47.5 $|-1\rangle$
	69.1 $|0\rangle$	66.9 $|0\rangle$	0.0 $|0\rangle$
	0.9 $|1\rangle$	1.7 $|1\rangle$	47.5 $|1\rangle$
	14.6 $|2\rangle$	14.8 $|2\rangle$	2.5 $|2\rangle$
3	43.2 $|-2\rangle$	43.5 $|-2\rangle$	2.5 $|-2\rangle$
	6.6 $|-1\rangle$	6.3 $|-1\rangle$	39.8 $|-1\rangle$
	0.3 $|0\rangle$	0.5 $|0\rangle$	15.5 $|0\rangle$
	6.6 $|1\rangle$	6.3 $|1\rangle$	39.8 $|1\rangle$
	43.2 $|2\rangle$	43.5 $|2\rangle$	2.5 $|2\rangle$
4	6.8 $|-2\rangle$	6.5 $|-2\rangle$	47.5 $|-2\rangle$
	42.4 $|-1\rangle$	41.7 $|-1\rangle$	2.5 $|-1\rangle$
	1.7 $|0\rangle$	3.6 $|0\rangle$	0.0 $|0\rangle$
	42.4 $|1\rangle$	41.7 $|1\rangle$	2.5 $|1\rangle$
	6.8 $|2\rangle$	6.5 $|2\rangle$	47.5 $|2\rangle$
5	34.6 $|-2\rangle$	33.7 $|-2\rangle$	34.7 $|-2\rangle$
	1.2 $|-1\rangle$	2.3 $|-1\rangle$	7.4 $|-1\rangle$
	28.5 $|0\rangle$	28.1 $|0\rangle$	15.8 $|0\rangle$
	1.2 $|1\rangle$	2.3 $|1\rangle$	7.4 $|1\rangle$
	34.6 $|2\rangle$	33.7 $|2\rangle$	34.7 $|2\rangle$

Table S5. Calculated magnetochemical parameters for the different iron(II) model structures of **1** and **2**. Parameters are reported according to the Blumberg notation.[a] Comparison between CASPT2 and NEVPT2 results.

	**1**HS		**1**HS@LS		**2**HS	
	CASPT2	NEVPT2	CASPT2	NEVPT2	CASPT2	NEVPT2
g_x	2.14	2.14	2.14	2.14	2.11	2.10
g_y	2.10	2.10	2.09	2.09	2.17	2.18
g_z	2.01	2.01	2.01	2.01	2.01	2.01
g_{av}	2.08	2.08	2.08	2.08	2.10	2.10
D / cm^{-1}	−6.58	−7.51	−6.65	−7.57	7.68	8.70
E / cm^{-1}	−1.60	−1.85	−1.48	−1.77	2.51	2.68
E/D[b]	0.24	0.25	0.22	0.23	0.33	0.31

[a] The Blumberg notation [19] (also known as the standard orientation) constrains the rhombicity to $0 \leq |E/D| \leq 1/3$ through appropriate axis permutation.

[b] For systems exhibiting near-maximal rhombicity ($|E/D| \approx 1/3$), the assignment of a specific magnetization direction (easy axis vs. easy plane) becomes physically ambiguous, as D values of opposite signs and different rhombicities can describe equivalent electronic states.

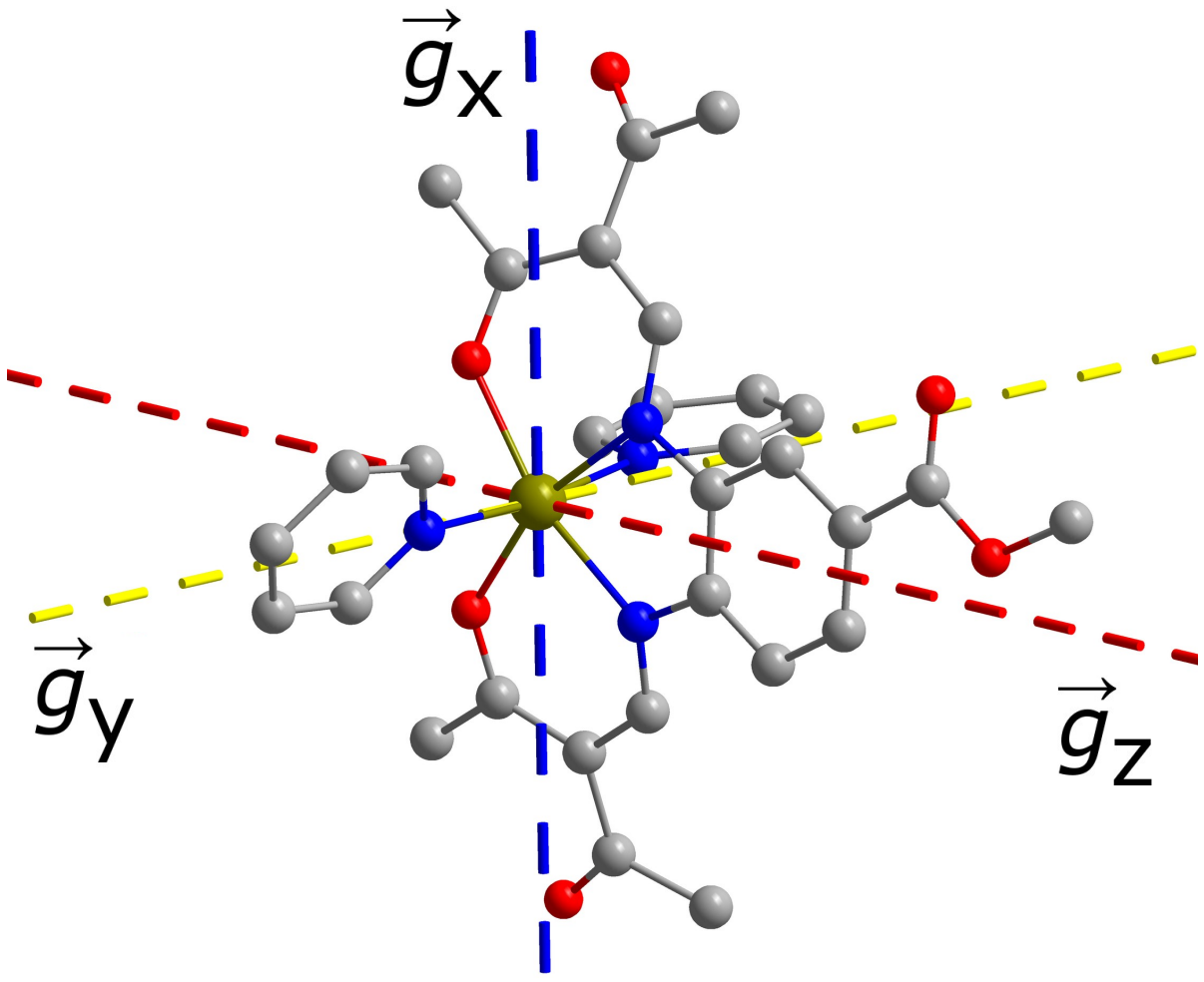

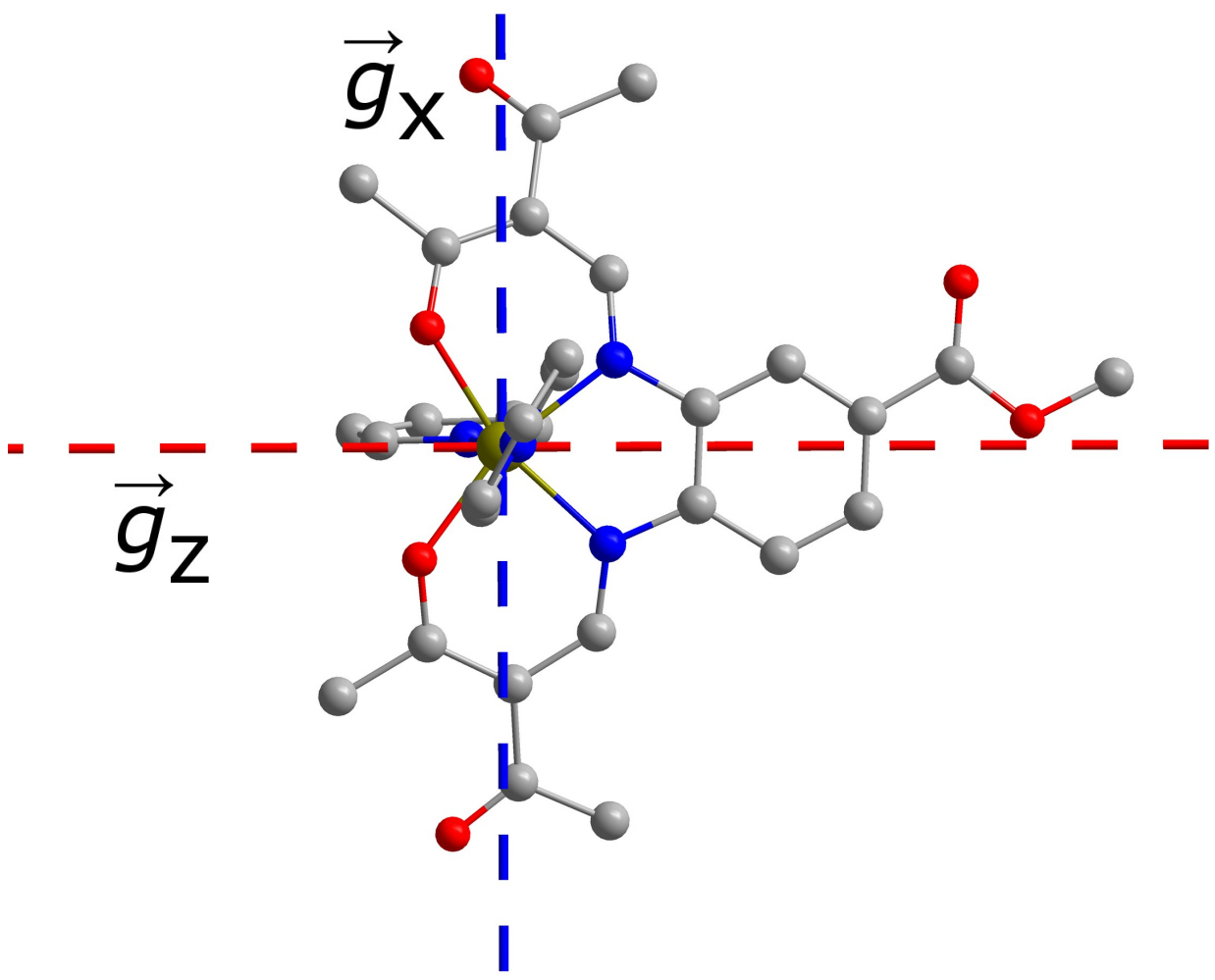

Figure S2. Orientation of the magnetic axes for **1**HS. (Top) Perspective view. (Bottom) View along the g_y axis. The g_x axis (blue) represents the easy axis of magnetization and is oriented approximately along the axis defined by the two opposite NO chelate pockets of the tetradentate ligand. Hydrogen atoms are omitted for clarity; the iron(II) ion is shown as an olive sphere.

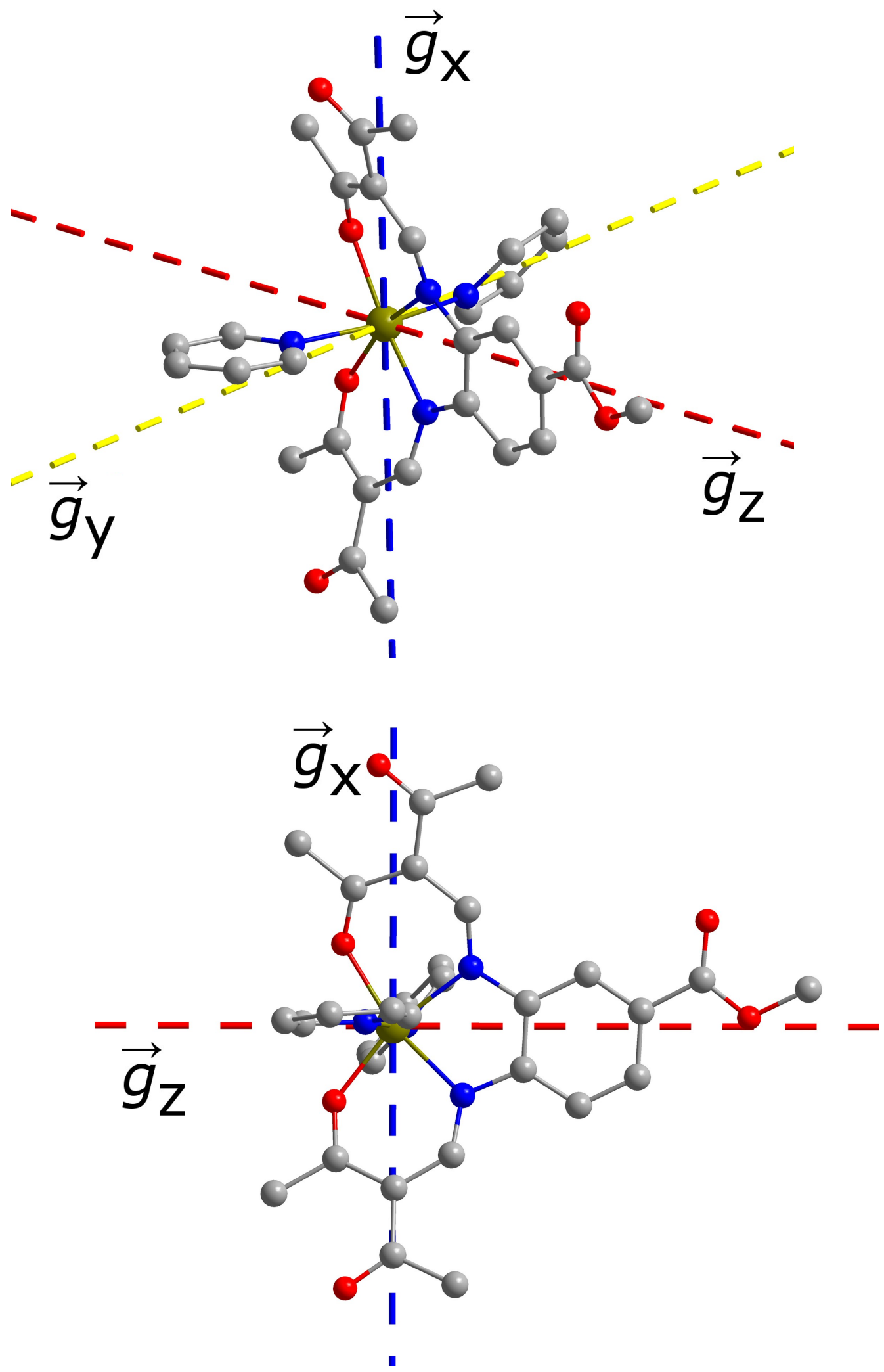

Figure S3. Orientation of the magnetic axes for **1**HS@LS. (Top) Perspective view. (Bottom) View along the g_y axis. The g_x axis (blue) represents the easy axis of magnetization and is oriented approximately along the axis defined by the two opposite NO chelate pockets of the tetradentate ligand. Hydrogen atoms are omitted for clarity; the iron(II) ion is shown as an olive sphere.

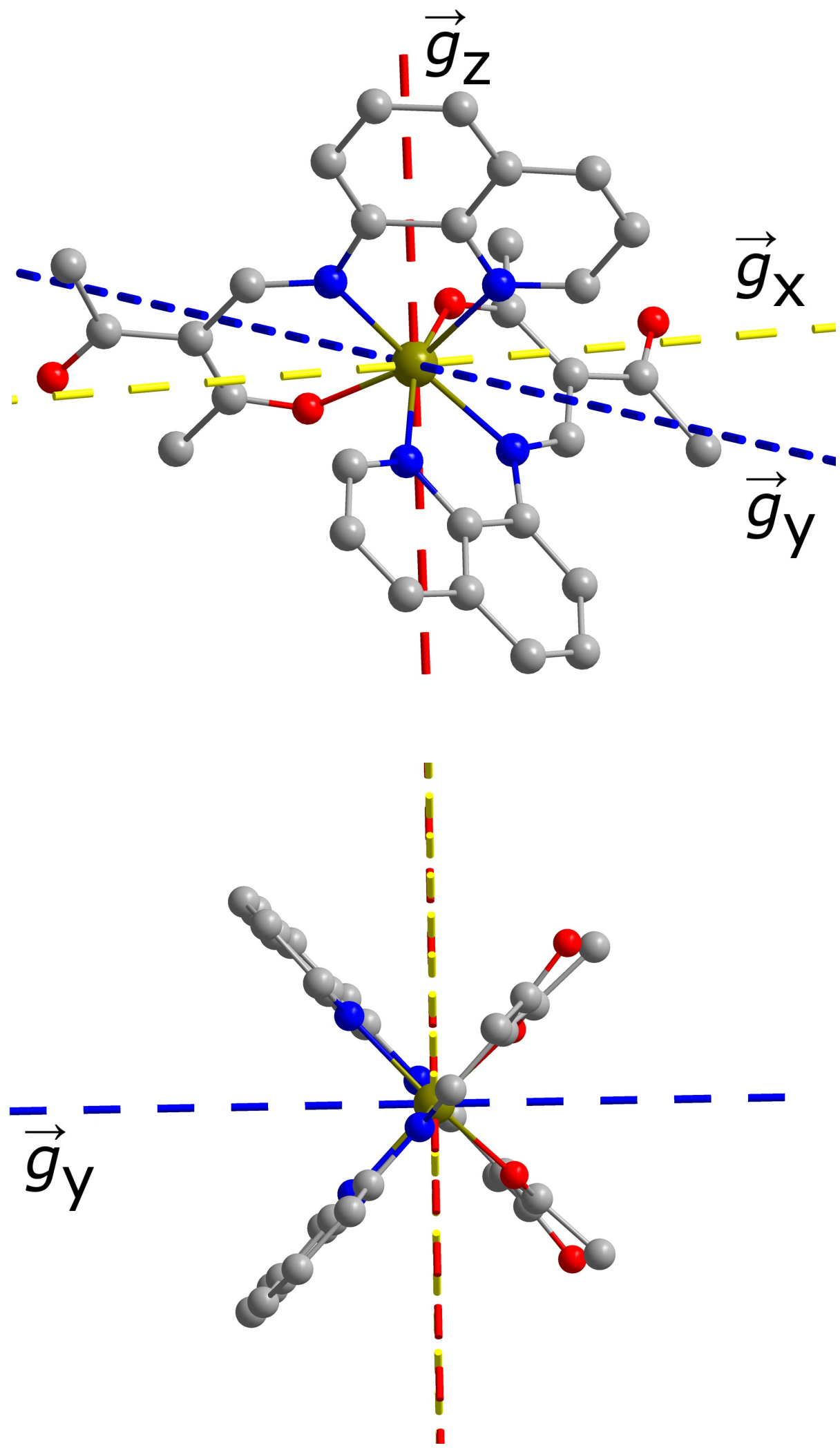

Figure S4. Orientation of the magnetic axes for **2**HS. (Top) Perspective view. (Bottom) View along the pseudo-fourfold axis approximately defined by the imine nitrogen donor atoms of the two tridentate ligands. This perspective illustrates that the mean planes of the tridentate chelate ligands possess a nearly 45° intersection angle with the plane defined by the g_x (yellow) and g_z (red) axes. It is also illustrated that the g_y axis (blue) lies within the N_2O_2 plane of the octahedral coordination sphere at the iron center, defined by the quinoline nitrogen and the two oxygen donors, bisecting the N···N and O···O edges. The g_y axis (blue) represents the easy axis of magnetization, while g_x (yellow) and g_z (red) represent the intermediate and hard axes, respectively. Hydrogen atoms are omitted for clarity; the iron(II) ion is shown as an olive sphere.

S2. THz-EPR Measurements

S2.1. Experimental

Low-temperature FD-FT THz-EPR measurements were carried out at the THz-EPR facility of BESSY II at Helmholtz-Zentrum Berlin (HZB). The spectrometer setup has been described previously.[20,21] It comprises a high-resolution FT-IR spectrometer (Bruker IFS 125 HR) equipped with selectable sources, among them a mercury lamp (unpolarized) and the synchrotron source (variable linear polarization), an evacuated optical transmission line, a 12 T superconducting magnet with a variable temperature insert (2–230 K), and a liquid-helium-cooled bolometer detector. Polycrystalline samples were mixed with polyethylene (PE) and pressed into pellets (**1** [Hg lamp]: 33.0 mg (76.0 mg PE); **1** [synchrotron]: 103.0 mg (32.0 mg PE); **2** [Hg lamp]: 28.6 mg (79.3 mg PE); **2** [synchrotron]: 61.9 mg (18.1 mg PE) and 51.5 mg (58.5 mg PE)). Spectra were recorded in Voigt geometry for magnetic fields $B_0 = 0$–10 T. Magnetic-field division spectra (MDS) were obtained by dividing spectra recorded at $B_0 + \Delta B$ by spectra recorded at B_0. Temperature-division spectra (TDS) were generated analogously by dividing spectra recorded at different temperatures. All temperature-dependent spectra were corrected for the PE background. The spectral resolution was set to 0.5 cm^{-1}. The wavenumber accuracy for well-resolved peaks is significantly higher and typically an order of magnitude better than the nominal resolution,[22] corresponding to <0.05 cm^{-1} under these conditions. Since the present analysis is based on relative shifts within a single measurement sequence, systematic errors are largely suppressed. Each spectrum represents an average of multiple scans. The digital data spacing was 0.015–0.03 cm^{-1} depending on the measurement conditions, providing sufficiently dense sampling for reliable peak determination. Peak positions were extracted from the spectral line shapes rather than individual data points, and relative peak shifts can thus be determined with higher precision than absolute energies. The resulting statistical uncertainty of peak positions is estimated to be 0.02–0.035 cm^{-1}, rendering the observed shifts clearly significant. Data analysis and simulations were performed using the EasySpin toolbox[23] within MATLAB.[24]

S2.2. Low-Lying Electronic Structure

For **1** and **2**, samples were quenched to 5 K (cooling rates 10–20 K / min) to obtain a maximally trapped *HS* fraction, hereafter denoted as the kinetically trapped *HS* state (*HS*-t). MDS were recorded at 5 K, and additional spectra at elevated temperatures (10 and 20 K) were used to construct TDS. The MDS of **2** exhibit six transitions at zero field, exceeding the four transitions expected for a ^{5}D multiplet (see Figure 4 in the main text). This arises because at 5 K the thermal energy is comparable to the energy splitting of the two lowest states (Figure S5), resulting in population of both levels and thus two sets of transitions. Accordingly, the observed signals can be grouped into three pairs with a constant energy offset corresponding to this splitting, enabling determination of the full multiplet structure. With increasing magnetic field, the transition energies follow the expected g-dependent shifts. At higher fields, line broadening and intensity reduction occur, consistent with the distribution of orientation-dependent effective g values in powder THz-EPR spectra. Deviations at higher energies are attributed to spin–phonon coupling (see below). For **1**, the overall spectral pattern is similar, but only three signals are resolved at zero field due to the smaller splitting of the lowest states and broader linewidths (see Figure S6). This is consistent with ab initio results (1 cm^{-1} for **1** vs. 3 cm^{-1} for **2**).

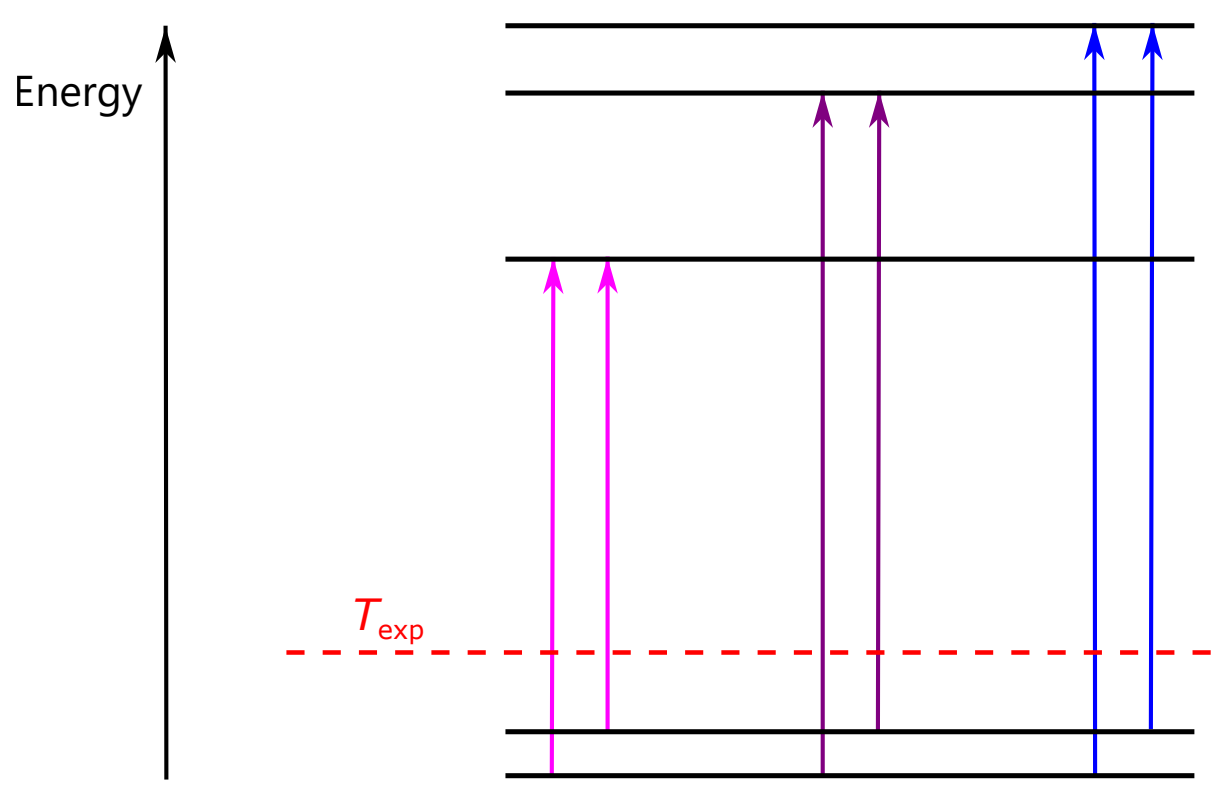

Figure S5. Schematic illustration of the five magnetic eigenstates for an $S = 2$ state resulting from axial and rhombic zero-field splitting. Owing to the low symmetry of the crystal field, m_S is not a strictly good quantum number and the eigenstates are mixed (Table S4). The red dashed line indicates the thermal energy at the experimental temperature (5 K); the two lowest eigenstates are significantly populated according to the Boltzmann distribution, giving rise to the six magnetic transitions marked by the magenta, purple, and blue arrows (see Table S6).

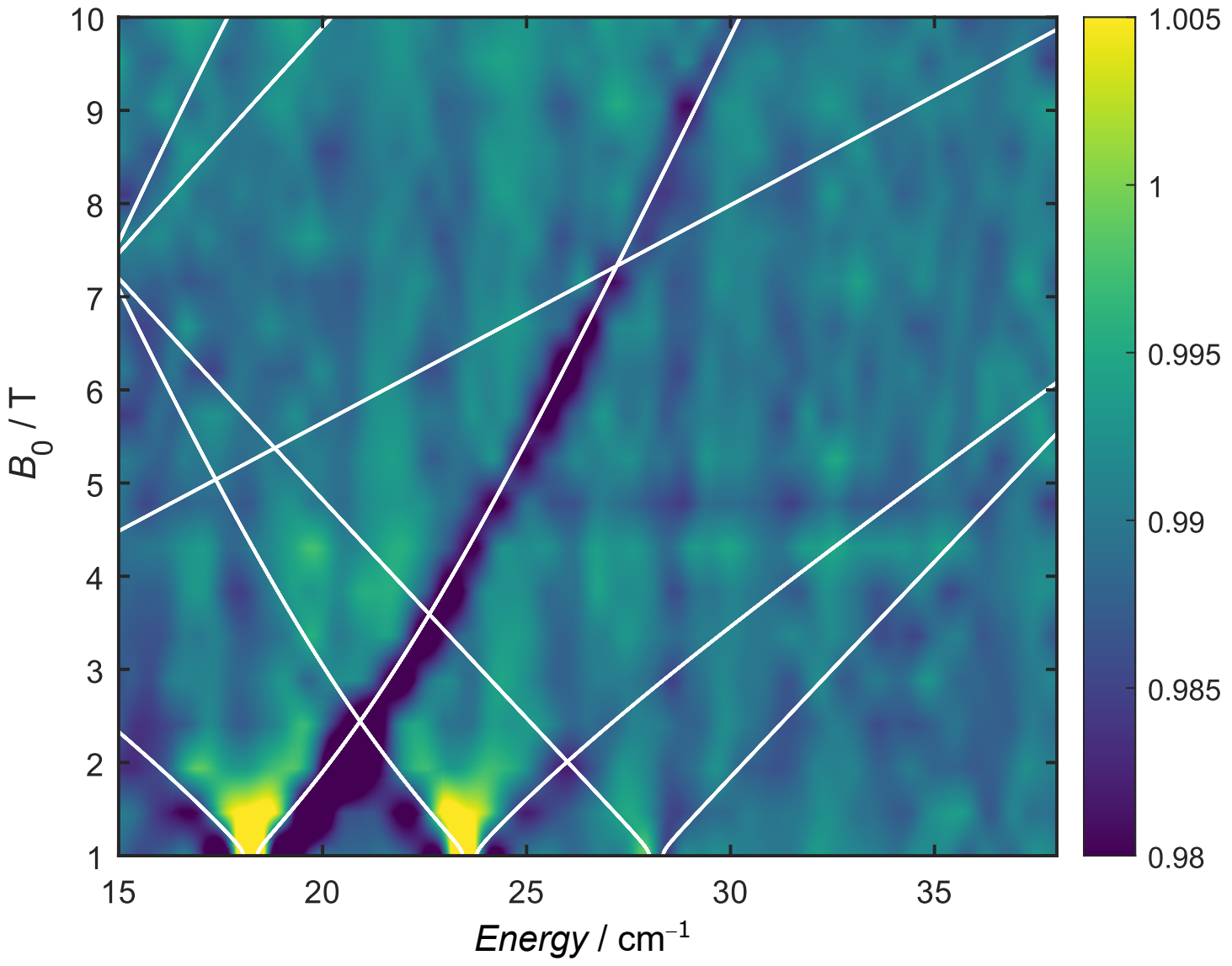

Figure S6. Contour plot of the magnetic field division spectra (MDS, $(B_0 + 0.5\,\mathrm{T})/B_0$, 5 K) of **1**. Relative intensity is color coded as indicated by the scale bar. Solid white lines show the simulated signal shifts from the best fit to Eq. (1) in the main text. Spectra were recorded using a mercury lamp as light source.

The assignment of the transitions of **2** and their relative intensities are summarized in Table S6. The intensity pattern is governed by the P-parity of the ZFS eigenstates: since the ZFS Hamiltonian commutes with $P : m_S \to -m_S$, the eigenstates carry definite P-parity (even or odd). The transition operators $\hat{S}_x$ (P-even) and $\hat{S}_y$ (P-odd) then impose selection rules: $\hat{S}_x$ connects states of the same parity, $\hat{S}_y$ connects states of different parity. Transitions $1 \to 3$ (even $\to$ even) and $2 \to 4$ (odd $\to$ odd) are therefore $\hat{S}_x$-allowed and are the most intense, while transitions $1 \to 4$, $2 \to 3$, and $2 \to 5$ are $\hat{S}_y$-allowed and carry intermediate intensity. Crucially, the transition $1 \to 5$ has *rigorously zero* intensity: both states belong to the P-even subspace $\{(|-2\rangle + |+2\rangle)/\sqrt{2}, |0\rangle\}$, within which $\hat{S}_x$ maps exclusively onto state 3, not state 5, while $\hat{S}_y = 0$ for same-parity states. The lower intensity of the '$2 \to$' transitions relative to '$1 \to$' transitions of the same type reflects the thermal population of state 2 according to the Boltzmann factor $e^{-\Delta E_{12}/k_B T}$ at $T = 5\,\mathrm{K}$, which is already encoded in the EasySpin simulation of Figure 4 of the main text.

Table S6. Assignment of the transitions observed in the MDS of **2** at 5 K. State numbers and zero-field energies from Table S3 (CASPT2). The dominant m_S character of each eigenstate is from Table S4; P-parity is derived therefrom (see text). Relative intensities are extracted from the EasySpin simulation already used to fit the MDS spectra (Figure 4 of main text, parameters from Table 1), re-run with reduced linewidth to resolve individual transitions; normalized to transition $1 \to 3$.[a]

Transition	P-parity ($i \to j$)	dom. operator	dom. m_S of i	dom. m_S of j	Rel. intensity
$1 \to 3$	even $\to$ even	$\hat{S}_x$	$\|0\rangle$	$\|\pm 1\rangle$	1.000
$2 \to 4$	odd $\to$ odd	$\hat{S}_x$	$\|\pm 1\rangle$	$\|\pm 2\rangle$	0.447
$2 \to 3$	odd $\to$ even	$\hat{S}_y$	$\|\pm 1\rangle$	$\|\pm 1\rangle$	0.442
$1 \to 4$	even $\to$ odd	$\hat{S}_y$	$\|0\rangle$	$\|\pm 2\rangle$	0.261
$2 \to 5$	odd $\to$ even	$\hat{S}_y$	$\|\pm 1\rangle$	$\|\pm 2\rangle/\|0\rangle$	0.061
$1 \to 5$	even $\to$ even	—	$\|0\rangle$	$\|\pm 2\rangle/\|0\rangle$	0[b]

[a] The EasySpin intensities at 5 K include Boltzmann population of both states at the respective gap energy; they are not simple multiples of the intrinsic (temperature-independent) transition matrix elements. Independent calculations with PHI[25] (using the intrinsic, temperature-unweighted matrix elements) confirm all qualitative trends: $1 \to 5$ is rigorously zero (numerical value $\lesssim 10^{-4}$ in PHI),transitions $1 \to 4$ agree quantitatively with EasySpin (ratio ≈ 1.00, confirming that differences for '$2 \to$' transitions arise from Boltzmann weighting only), and PHI($2 \to 3$) = PHI($2 \to 4$) for the experimental fit parameters, providing direct confirmation of the P-parity selection rule.

[b] Symmetry-forbidden: both states belong to the same P-even subspace; neither $\hat{S}_x$ (which maps this subspace onto state 3 only) nor $\hat{S}_y$ (zero for same-parity states) can connect them.

[c] The dominant m_S character of each eigenstate is assigned on the basis of the percentage projections given in Table S4.

S2.3. Spin–Phonon Coupling

For **2**, additional spectral features above 30 cm^{-1} cannot be reproduced by a pure spin Hamiltonian. In particular, signals at 33 and 40 cm^{-1} exhibit field-dependent intensity changes and shifts characteristic of avoided crossings with vibrational modes, as evidenced by the TDS (see Figure 4 in the main text). The intensity of these features initially increases with magnetic field, followed by broadening and a gradual loss of intensity at higher fields. This behavior is consistent with spin–phonon coupling and can be described using an empirical coupling Hamiltonian given in (S1),[26,27] where E_{sp}, E_{ph1}, and E_{ph2} are the energies of the magnetic and phonon excitations, respectively, and Λ_1 and Λ_2 are the spin–phonon coupling constants ($\Lambda_1 = 0.8$ and $\Lambda_2 = 0.6$).

$$\widehat{H} = \begin{bmatrix} E_{sp} & \Lambda_1 & \Lambda_2 \\ \Lambda_1 & E_{ph1} & 0 \\ \Lambda_2 & 0 & E_{ph2} \end{bmatrix} \tag{S1}$$

The resulting simulations (see Figs. 4 and 5 in the main text) reproduce the experimental features well.

S2.4. Temperature- and Time-Dependent Measurements

Two complementary measurement protocols were employed.

(i) The temperature cycle described in the main text, comprising both temperature- and time-dependent elements, was applied to the same samples used for the field-dependent THz-EPR measurements. This protocol includes MDS and TDS at low temperatures as well as time-resolved measurements at elevated temperatures (without magnetic field variation).

(ii) In addition, independent temperature-dependent series were recorded on separate samples to systematically investigate the thermal evolution of the spectra under zero magnetic field conditions.

S2.4.1. Time-Dependent Measurements at Elevated Temperatures

Time-dependent THz measurements were performed at elevated temperatures to monitor the relaxation dynamics of the trapped *HS*-t state. In contrast to the low-temperature field-dependent THz-EPR experiments, these measurements were carried out at zero magnetic field on the same samples as used in the main text. For both compounds, spectra were recorded consecutively at fixed temperatures following a controlled heating protocol. The selected temperature ranges correspond to the regime where thermally activated spin crossover occurs on experimentally accessible timescales. For **1**, time-dependent spectra were recorded at 100 K using synchrotron radiation. For **2**, analogous measurements were performed in the temperature range 90–100 K using a mercury lamp; for the analysis shown in Figure S7, the series recorded at 90 K was used. To visualize the temporal evolution, time-division spectra (tDS) were constructed by dividing consecutive spectra within each series. The resulting tDS directly reflect changes in spectral intensity over time and thus provide access to the relaxation of the kinetically trapped *HS*-t fraction. In both systems, the tDS reveal a gradual decrease in intensity of features associated with magnetic excitations, indicating the progressive conversion of *HS*-t centers into the relaxed *LS*-r state. The continuous nature of these changes supports a thermally activated relaxation process on the timescale of the experiment.

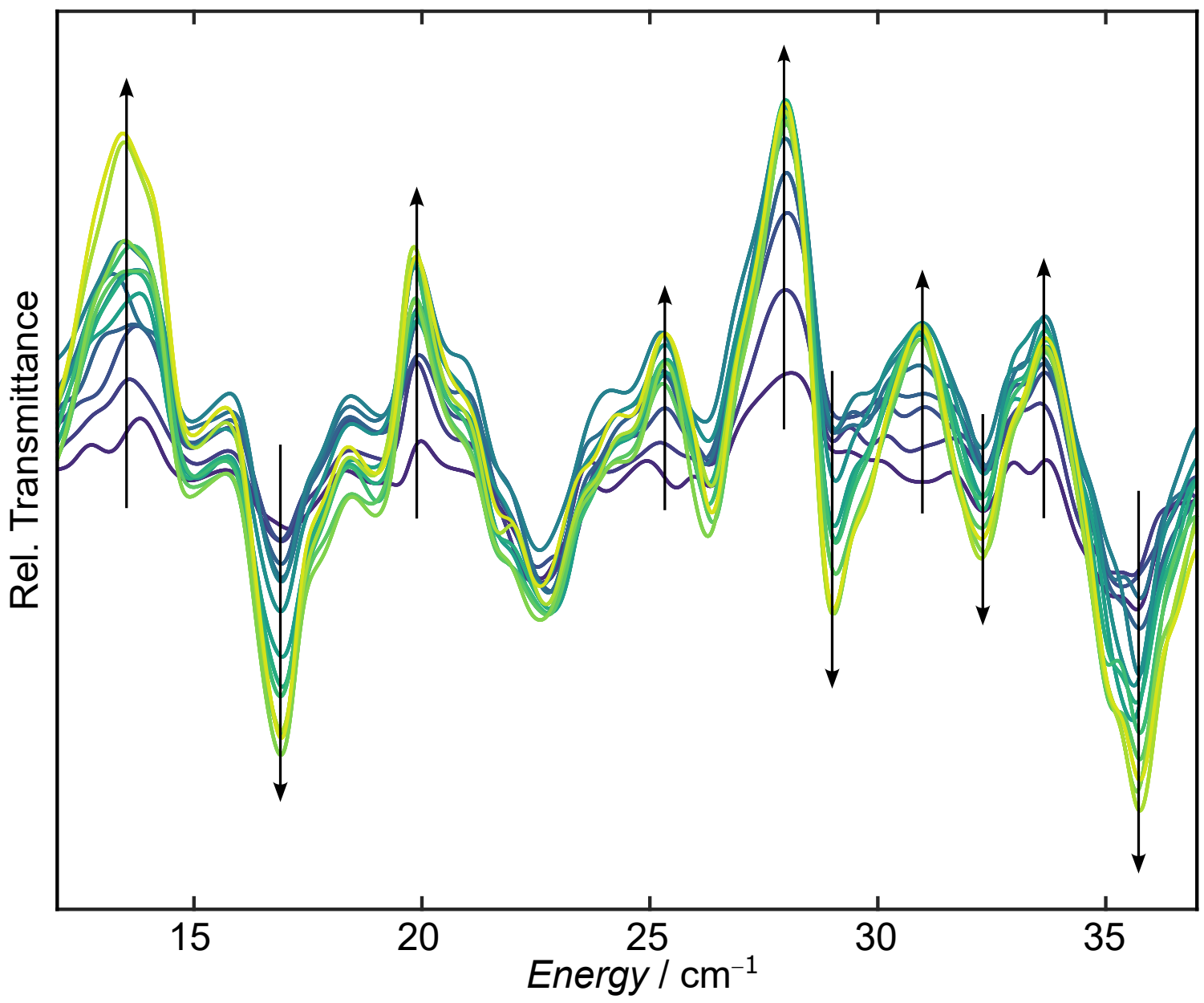

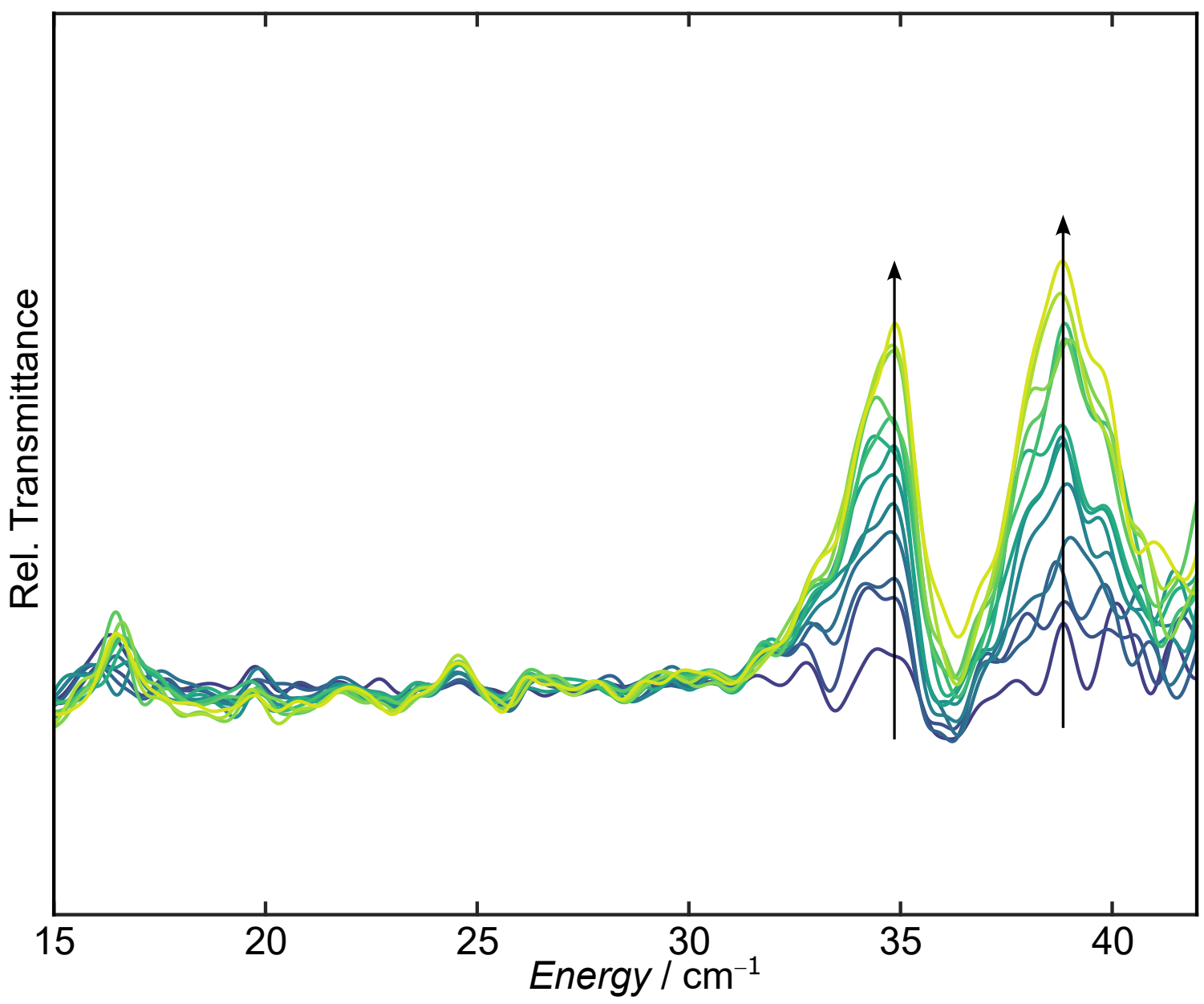

Figure S7. Time-division spectra (tDS) illustrating the temporal evolution of the THz signal at elevated temperatures. Top: **1** at 100 K (4 min per spectrum). Bottom: **2** at 90 K (1 min per spectrum). The color scale represents increasing measurement time from dark blue to yellow. Arrows highlight the evolution of selected spectral features.

S2.4.2. Comparison of *HS*-t and *LS*-r States at 5 K

To establish spectroscopic fingerprints of the spin states, THz spectra of the trapped high-spin state (*HS*-t) and the relaxed low-spin state (*LS*-r) were compared at 5 K. The *LS*-r state was obtained by thermal relaxation at elevated temperature followed by stepwise cooling to 5 K within the independent temperature-dependent measurement protocol described below (see Section S2.4.3). At low temperatures, comparing the THz spectra of the *HS*-t and *LS*-r states at 5 K (Figure S8) reveals pronounced intensity variations (peak positions are indicated in Figure S8 and are not further quantified here, as the analysis focuses on relative changes). The most significant reductions in the *LS*-r state occur for signals that coincide in energy with the magnetic excitations previously identified via MDS (specifically peaks D and E for **1**; A, B, C, and D for **2**), indicating a significant contribution of magnetic transitions. In addition, several signals (e.g., peaks B, C, F, G, and H for **1**; E and G for **2**) exhibit distinct energetic shifts and intensity changes that cannot be solely attributed to magnetic transitions. These observations point toward modifications of the lattice dynamics upon spin crossover. In particular, the appearance of mode F for **2** in the *LS*-r state highlights changes in the vibrational spectrum. These findings are consistent with the lattice-induced modifications of the *HS* electronic structure discussed in the main text, indicating that structural relaxation affects both vibrational modes and the electronic environment of residual *HS* centers.

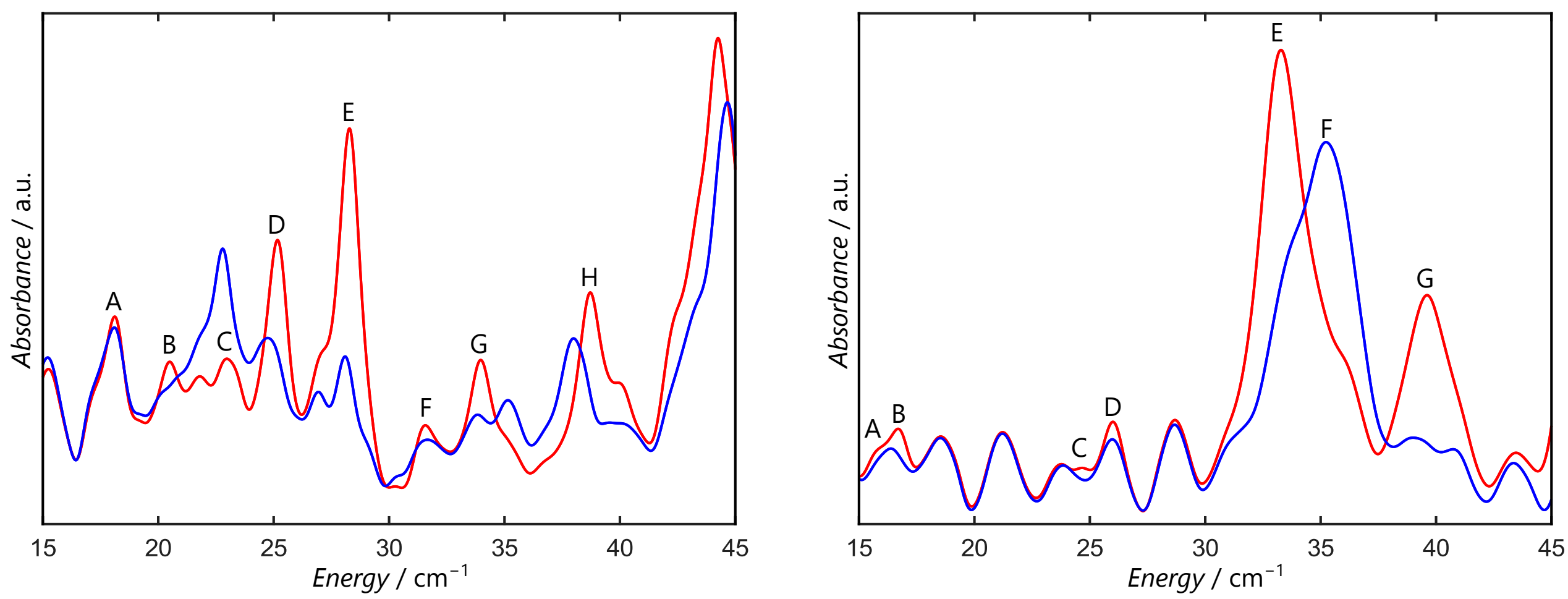

Figure S8. THz spectra of **1** (left) and **2** (right) measured at 5 K for the trapped high-spin state (*HS*-t, red, obtained by rapid cooling) and the relaxed low-spin state (*LS*-r, blue, obtained after thermal relaxation and recooling). The letters mark the features/peaks that change upon spin crossover.

S2.4.3. Temperature-Dependent Measurements

To complement the measurements discussed above, additional temperature-dependent THz spectra were recorded on separate samples using a modified temperature protocol without application of an external magnetic field. Samples were rapidly cooled to 5 K at rates of 10–20 K / min to maximize the trapped *HS*-t fraction and subsequently measured upon heating from 5 to 150 K (5 and 20 K, followed by 10 K steps between 40 and 150 K) and upon cooling using the same sequence. In contrast to the previously described measurements, no magnetic-field dependent spectra were recorded in this series.

These measurements reveal three characteristic regimes:

- trapped high-spin state at low temperatures (*HS*-t),
- relaxed low-spin state at intermediate temperatures (*LS*-r),
- thermally equilibrated high-spin state at higher temperatures (*HS*-r).

The resulting spectral evolution is shown in Figures S9–S12. The data are presented both as color-coded plots (Figures S9 and S10) and as stacked spectra (Figures S11 and S12), allowing visualization of both continuous spectral changes and individual peak evolution.

Upon heating from 5 K, both compounds exhibit a gradual decrease in the intensity of features associated with the *HS*-t state. While a reduction in the intensity of purely magnetic excitations is generally expected due to thermal population redistribution according to Boltzmann statistics, the simultaneous disappearance of specific *HS* vibrational modes reflects the actual structural relaxation of the trapped *HS*-t fraction into the *LS*-r state.

In the intermediate temperature range, the spectra are dominated by the vibrational signature of the relaxed *LS*-r configuration. At these temperatures, the *HS*-specific spin–phonon coupled modes and purely magnetic contributions are entirely absent, which is consistent with the diamagnetic ($S = 0$) nature of the iron(II) *LS* state. At higher temperatures, the re-emergence of these spectral features indicates the repopulation of the thermally equilibrated *HS* state (*HS*-r), consistent with the expected SCO equilibrium at elevated temperatures. Upon subsequent cooling, the spectral evolution differs from the heating branch. Instead of recovering the initial *HS*-t state, the system evolves toward the relaxed *LS*-r state at low temperatures, confirming that the trapped *HS*-t configuration is a non-equilibrium state accessible only via rapid cooling. In addition to intensity changes, several modes exhibit small but systematic energy shifts throughout the temperature cycle. These shifts reflect modifications of the vibrational and electronic environment upon SCO and are consistent with the lattice-induced effects on the *HS* electronic structure discussed in the main text. Overall, these independent temperature-dependent measurements confirm the coexistence of three characteristic regimes: the trapped *HS*-t state at low temperatures, the relaxed *LS*-r state at intermediate temperatures, and the thermally equilibrated *HS*-r state at higher temperatures.

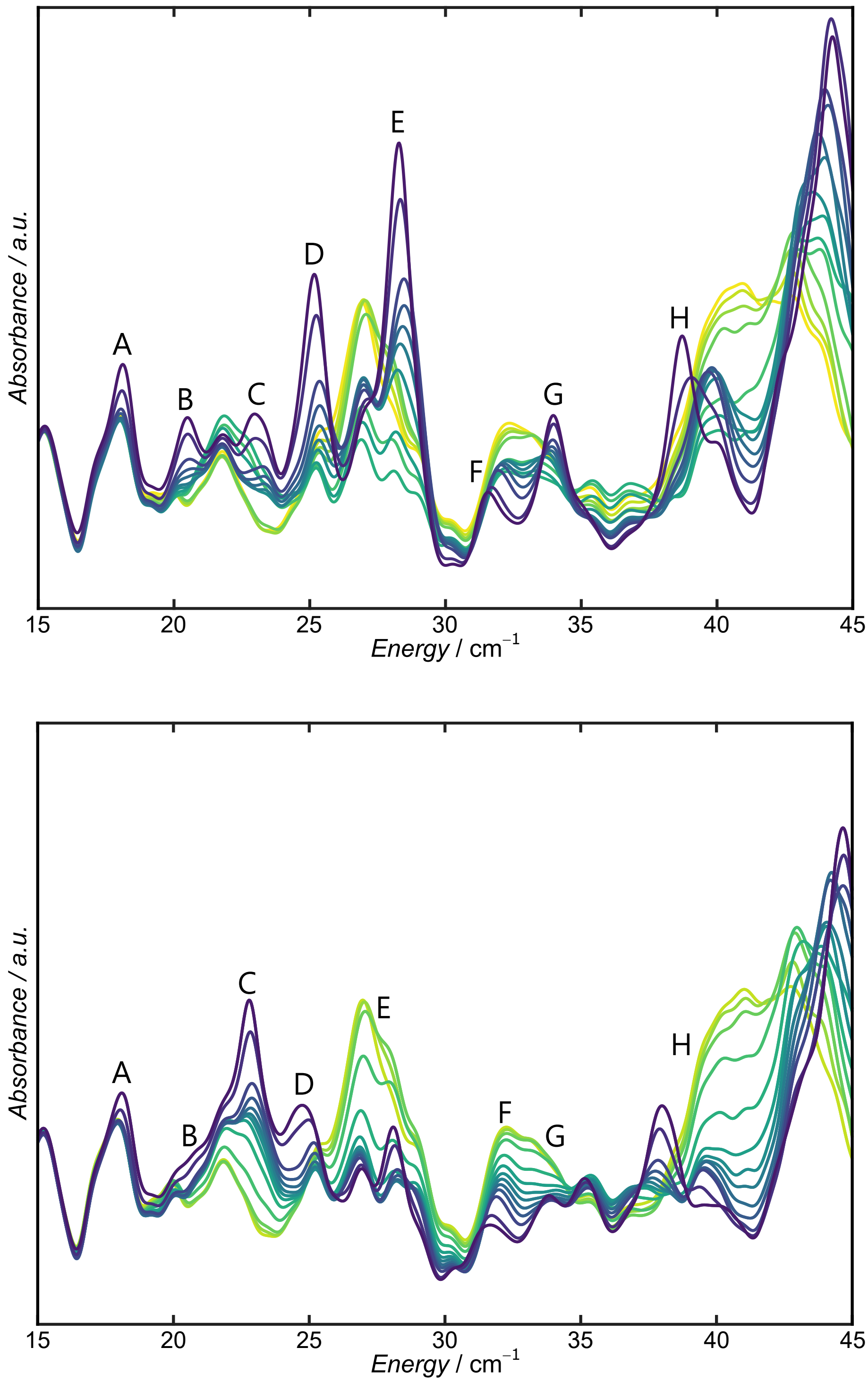

Figure S9. Temperature dependence of the THz spectra of **1** at 0 T. The temperature is color coded from dark violett over green to yellow , corresponding to an increase from 5 K to 150 K. Top: Heating sequence starting from the kinetically trapped *HS* state (*HS*-t) at 5 K, passing through a relaxed *LS* state (*LS*-r), and reaching the thermally equilibrated *HS* state (*HS*-r) at elevated temperature (TIESST experiment). Bottom: Subsequent cooling, showing the evolution toward the relaxed *LS*-r state at 5 K.

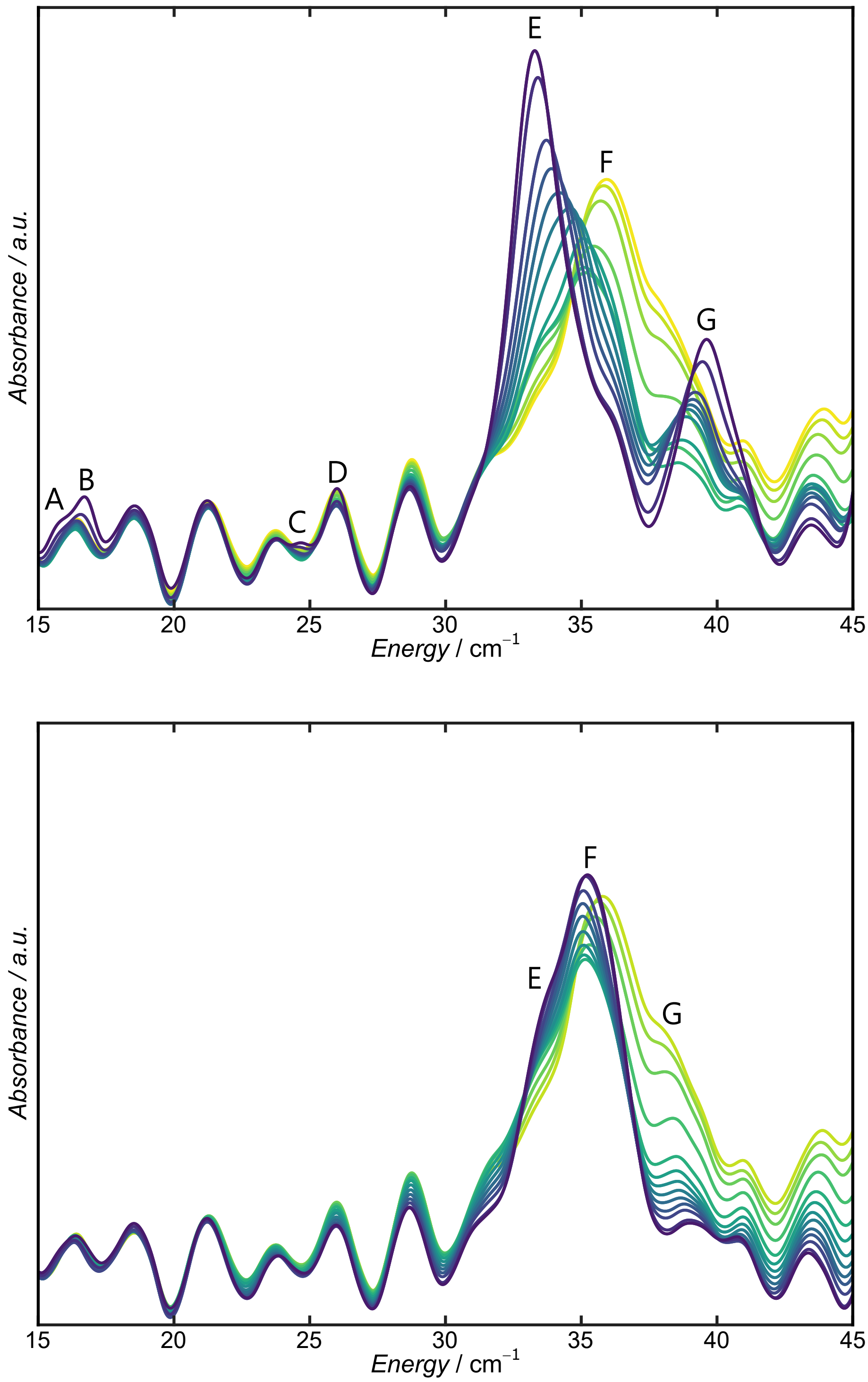

Figure S10. Temperature dependence of the THz spectra of **2** at 0 T. The temperature is color coded from dark violett over green to yellow , corresponding to an increase from 5 K to 150 K. Top: Heating sequence starting from the kinetically trapped *HS* state (*HS*-t) at 5 K, passing through a relaxed *LS* state (*LS*-r), and reaching the thermally equilibrated *HS* state (*HS*-r) at elevated temperature (TIESST experiment). Bottom: Subsequent cooling, showing the evolution toward the relaxed *LS*-r state at 5 K.

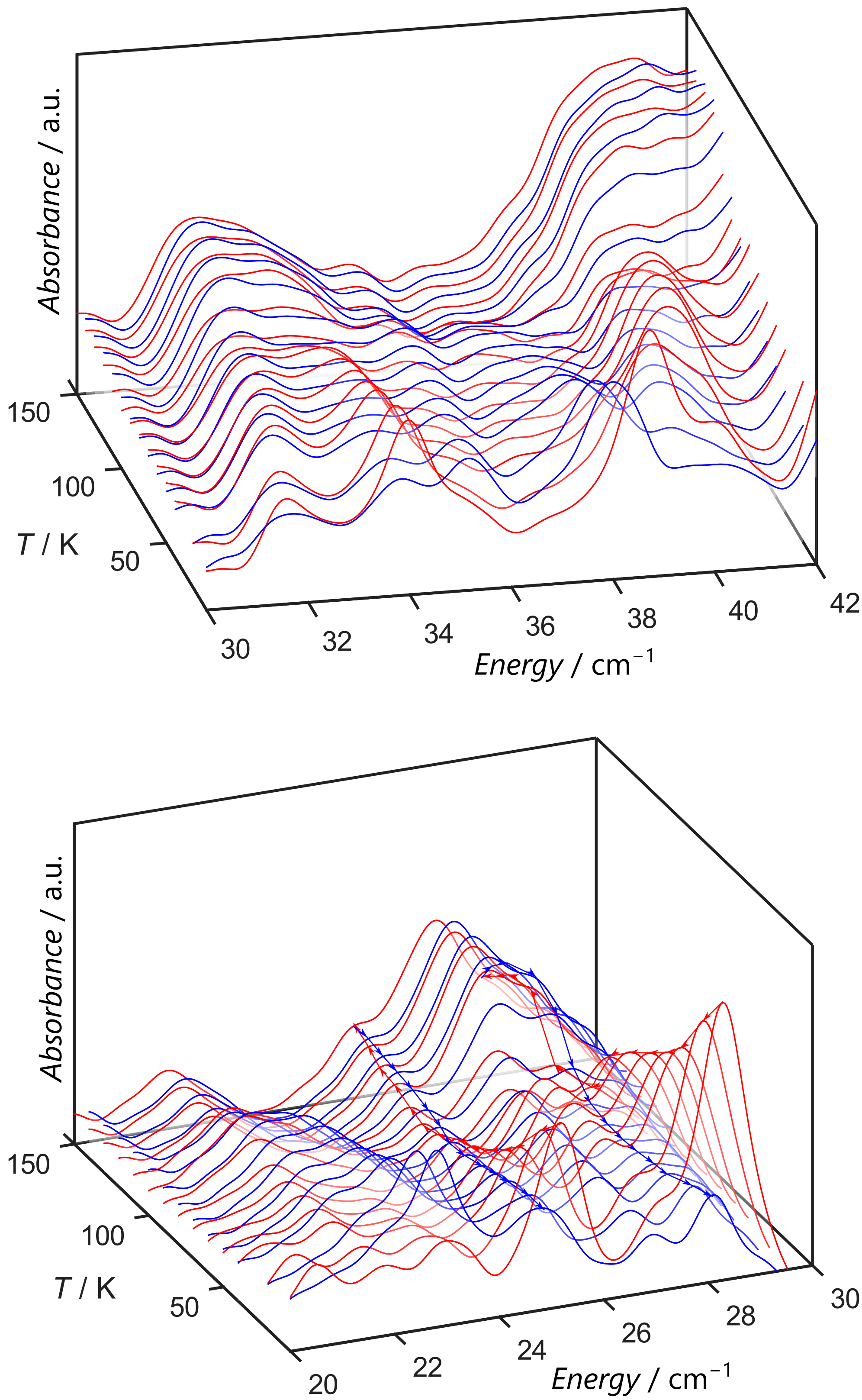

Figure S11. Temperature-dependent THz spectra of **1** at 0 T. Top: 20–30 cm^{-1}; bottom: 30–50 cm^{-1}. Red traces correspond to measurements recorded upon heating from the kinetically trapped *HS* state (*HS*-t) at 5 K to the thermally equilibrated *HS* state at 150 K, passing through an intermediate relaxed *LS* state (*LS*-r) (TIESST experiment). Blue traces show the subsequent cooling, leading to the relaxed *LS* state (*LS*-r) at 5 K. Red and blue arrows are included as guides to the eye to illustrate the measurement sequence.

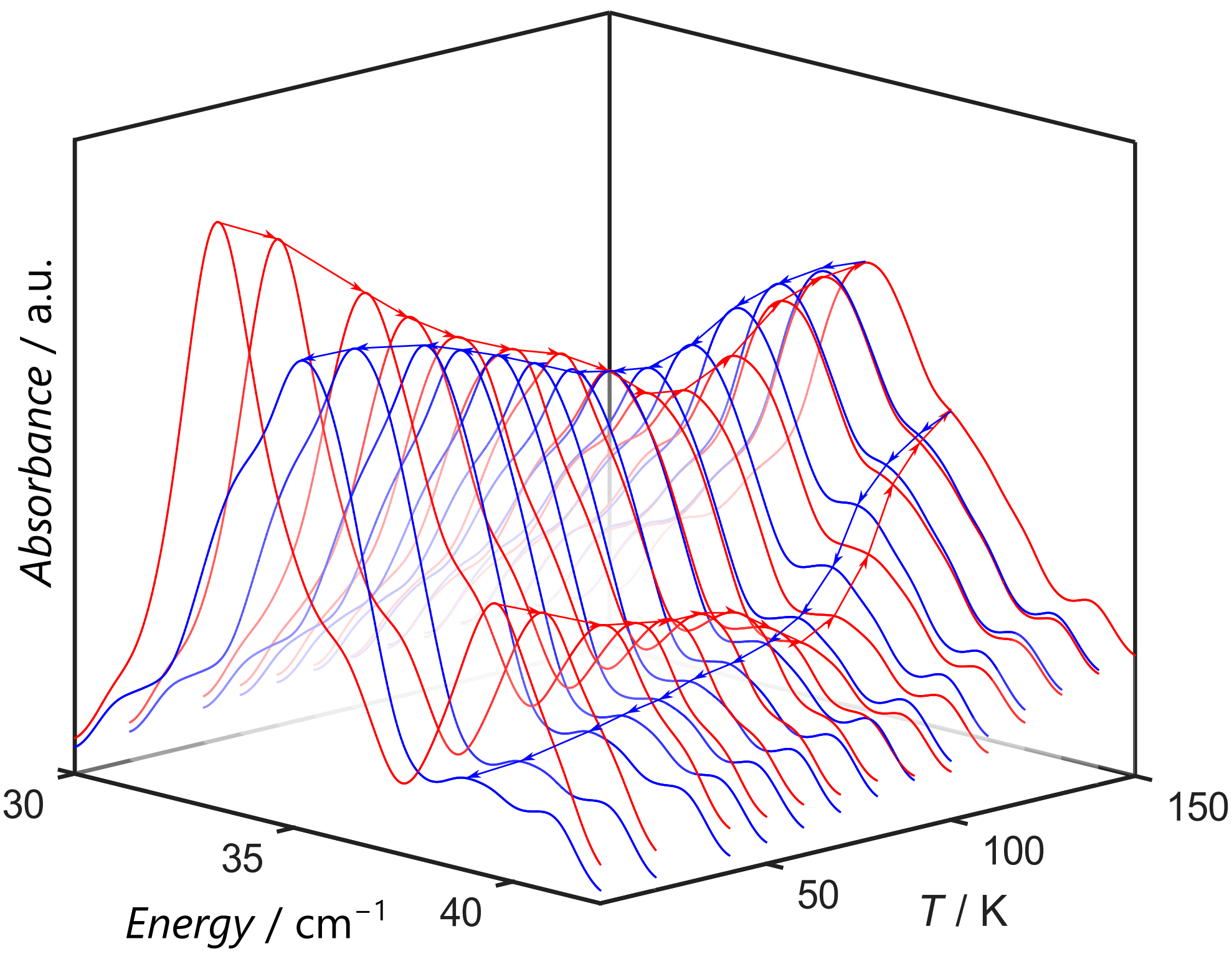

Figure S12. Temperature-dependent THz spectra of **2** at 0 T. Red traces correspond to measurements recorded upon heating from the kinetically trapped *HS* state (*HS*-t) at 5 K to the thermally equilibrated *HS* state at 150 K, passing through an intermediate relaxed *LS* state (*LS*-r) (TIESST experiment). Blue traces show the subsequent cooling, leading to the relaxed *LS* state (*LS*-r) at 5 K. Red and blue arrows are included as guides to the eye to illustrate the measurement sequence.

S2.5. Field-Dependent MDS of the Post-SCO State of Compound 1

Field-dependent MDS of compound **1** were also recorded after thermally induced spin crossover (post-SCO state, LS-r matrix with residual HS fraction $\gamma_{HS} \approx 0.5$), over the range 0–4 T in 0.5 T steps. Independent fitting to Eq. (1) yields the post-state parameters summarized in Table S7, with the spectra and fit shown in Figure S13. The comparison demonstrates a small decrease in rhombicity ($\Delta|E| = -0.069\,\text{cm}^{-1}$) at essentially unchanged $|D|$, consistent in sign and order of magnitude with the *ab initio* prediction for the 1HS→1HS@LS structural transition (Table S5).

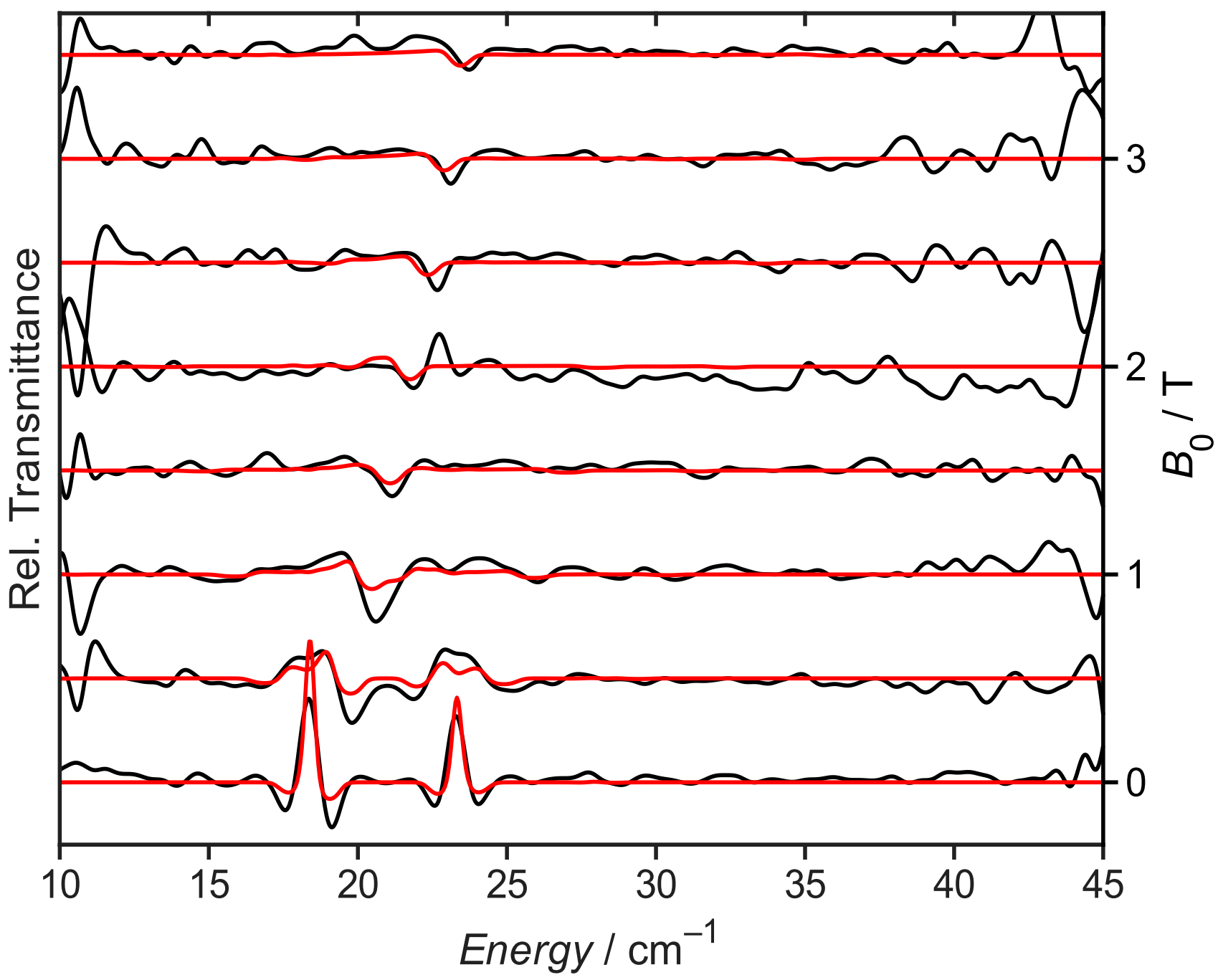

Figure S13. Magnetic-field division spectra (MDS, $(B_0{+}0.5\,\text{T})/B_0$, 5 K) of **1** after thermally induced spin crossover (LS-r state, $\gamma_{HS} \approx 0.5$), recorded over the field range 0–4 T in 0.5 T steps. Black lines: experimental data; red lines: best fit to Eq. (1) of the main text with parameters $g = 1.94$, $|D| = 6.915\,\text{cm}^{-1}$, $|E| = 0.827\,\text{cm}^{-1}$. Spectra were recorded using a mercury lamp as light source.

Table S7. ZFS parameters for compound **1** in the kinetically trapped HS state (ante, HS-t) and after thermally induced spin crossover (post, LS-r), compared with the *ab initio* prediction for the 1HS→1HS@LS structural transition (CASPT2, Table S5).

	Ante (HS-t)	Post (LS-r)	Δ(exp)	Δ(ab initio)
g	2.088	1.937[a]	−0.151[a]	≈ 0
$\|D\|$ / cm^{-1}	6.915	6.915	≈ 0	−0.07
$\|E\|$ / cm^{-1}	0.896	0.827	−0.069	−0.12[b]
E/D	0.130	0.120	−0.010	−0.02

[a] The change in g in the post-state fit is attributed to the limited field range of the post-state data (0–4 T vs. 0–9 T for the ante state), which constrains the determination of g less well.

[b] *Ab initio* $\Delta|E| = -0.12\,\text{cm}^{-1}$: $|E|$ decreases from 1.60 to 1.48 cm^{-1} in the 1HS→1HS@LS structural transition (CASPT2, Table S5), same direction as the experimental observation.

References

[1] F. Daumann, G. Hörner, P. Köhler, B. Weber, "Half the Step, Twice the Mystery–Kinetically Controlled Spin Crossover in an Anticooperative Pyridine-Coordinate JÄGER-Type Iron(II) Complex," *Crystal Growth & Design* 25 (2025): 4565–4576, https://doi.org/10.1021/acs.cgd.5c00477.

[2] A. Dürrmann, G. Hörner, B. Weber, "Remote H/F Tuning of Cooperativity in $(NN'O)_2$-Coordinate Iron(II) Complexes: Dynamic, Hysteretic, and Continuous Spin Crossover," *Crystal Growth & Design* 23 (2023): 1743–1754, https://doi.org/10.1021/acs.cgd.2c01287.

[3] "TURBOMOLE V7.8 2023, a development of University of Karlsruhe and Forschungszentrum Karlsruhe GmbH, 1989-2007, TURBOMOLE GmbH, since 2007; available from https://www.turbomole.org,".

[4] S. G. Balasubramani, G. P. Chen, S. Coriani et al., "TURBOMOLE: Modular program suite for ab initio quantum-chemical and condensed-matter simulations," *The Journal of Chemical Physics* 152 (2020): 184107, https://doi.org/10.1063/5.0004635.

[5] E. J. Baerends, D. E. Ellis, P. J. C. P. Ros, "Self-consistent molecular Hartree—Fock—Slater calculations I. The computational procedure," *Chemical Physics* 2 (1973): 41–51, https://doi.org/10.1016/0301-0104(73)80059-X.

[6] B. I. Dunlap, J. W. D. Connolly, J. R. Sabin, "On first-row diatomic molecules and local density models," *The Journal of Chemical Physics* 71 (1979): 4993–4999, https://doi.org/10.1063/1.438313.

[7] C. Van Alsenoy, "Ab initio calculations on large molecules: The multiplicative integral approximation," *Journal of Computational Chemistry* 9 (1988): 620–626, https://doi.org/10.1002/jcc.540090607.

[8] J. L. Whitten, "Coulombic potential energy integrals and approximations," *The Journal of Chemical Physics* 58 (1973): 4496–4501, https://doi.org/10.1063/1.1679012.

[9] C. Adamo, V. Barone, "Toward reliable adiabatic connection models free from adjustable parameters," *Chemical Physics Letters* 274 (1997): 242–250, https://doi.org/10.1016/S0009-2614(97)00651-9.

[10] C. Adamo, V. Barone, "Toward reliable density functional methods without adjustable parameters: The PBE0 model," *The Journal of Chemical Physics* 110 (1999): 6158–6170, https://doi.org/10.1063/1.478522.

[11] F. Weigend, "Accurate Coulomb-fitting basis sets for H to Rn," *Physical Chemistry Chemical Physics* 8 (2006): 1057–1065, https://doi.org/10.1039/B515623H.

[12] G. L. Manni, I. F. Galván, A. Alavi et al., "The OpenMolcas *Web*: A Community-Driven Approach to Advancing Computational Chemistry," *Journal of Chemical Theory and Computation* 19 (2023): 6933–6991, https://doi.org/10.1021/acs.jctc.3c00182.

[13] J. P. Zobel, P.-O. Widmark, V. Veryazov, "The ANO-R basis set," *Journal of Chemical Theory and Computation* 16 (2019): 278–294, https://doi.org/10.1021/acs.jctc.9b00873.

[14] J. P. Finley, "Similarities between single reference perturbation theory based on a CASSCF wavefunction and multireference perturbation theory based on a reference space spanned by a CAS," *Chemical Physics Letters* 283 (1998): 277–282, https://doi.org/10.1016/s0009-2614(97)01410-3.

[15] P. Å. Malmqvist, B. O. Roos, B. Schimmelpfennig, "The restricted active space (RAS) state interaction approach with spin–orbit coupling," *Chemical Physics Letters* 357 (2002): 230–240, https://doi.org/10.1016/S0009-2614(02)00498-0.

[16] B. A. Heß, C. M. Marian, U. Wahlgren, O. Gropen, "A mean-field spin-orbit method applicable to correlated wavefunctions," *Chemical Physics Letters* 251 (1996): 365–371, https://doi.org/10.1016/0009-2614(96)00119-4.

[17] L. F. Chibotaru, L. Ungur, "*Ab initio* calculation of anisotropic magnetic properties of complexes. I. Unique definition of pseudospin Hamiltonians and their derivation," *The Journal of Chemical Physics* 137 (2012): 064112, https://doi.org/10.1063/1.4739763.

[18] F. Neese, "Software update: The ORCA program system—version 6.0," *Wiley Interdisciplinary Reviews: Computational Molecular Science* 15 (2025): e70019, https://doi.org/10.1002/wcms.70019.

[19] W. E. Blumberg, "Nuclear Spin-Lattice Relaxation Caused by Paramagnetic Impurities," *Physical Review* 119 (1960): 79–84, https://doi.org/10.1103/PhysRev.119.79.

[20] J. Nehrkorn, K. Holldack, R. Bittl, A. Schnegg, "Recent progress in synchrotron-based frequency-domain Fourier-transform THz-EPR," *Journal of Magnetic Resonance* 280 (2017): 10–19, https://doi.org/10.1016/j.jmr.2017.04.001.

[21] A. Schnegg, J. Behrends, K. Lips, R. Bittl, K. Holldack, "Frequency domain Fourier transform THz-EPR on single molecule magnets using coherent synchrotron radiation," *Physical Chemistry Chemical Physics* 11 (2009): 6820–6825, https://doi.org/10.1039/B905745E.

[22] S. T. Nicolau, A. J. Matzger, "An evaluation of resolution, accuracy, and precision in FT-IR spectroscopy," *Spectrochimica Acta Part A: Molecular and Biomolecular Spectroscopy* 319 (2024): 124545, https://doi.org/10.1016/j.saa.2024.124545.

[23] S. Stoll, A. Schweiger, "EasySpin, a comprehensive software package for spectral simulation and analysis in

EPR," *Journal of Magnetic Resonance* 178 (2006): 42–55, https://doi.org/10.1016/j.jmr.2005.08.013.
[24] The MathWorks Inc., "MATLAB," **2022**.
[25] N. F. Chilton, R. P. Anderson, L. D. Turner, A. Soncini, K. S. Murray, "PHI: A powerful new program for the analysis of anisotropic monomeric and exchange-coupled polynuclear *d*- and *f*-block complexes," *J. Comput. Chem.* 34 (2013): 1164–1175, https://doi.org/10.1002/jcc.23234.
[26] M. H. Pohle, M. Böhme, T. Lohmiller et al., "Magnetic Anisotropy and Relaxation of Pseudotetrahedral [N_2O_2] Bis-Chelate Cobalt(II) Single-Ion Magnets Controlled by Dihedral Twist Through Solvomorphism," *Chemistry—A European Journal* 29 (2023): e202202966, https://doi.org/10.1002/chem.202202966.
[27] D. H. Moseley, S. E. Stavretis, K. Thirunavukkuarasu et al., "Spin-phonon couplings in transition metal complexes with slow magnetic relaxation," *Nature Communications* 9 (2018): 2572, https://doi.org/10.1038/s41467-018-04896-0.
